# The genetic interaction network of *CCW12*, a *Saccharomyces cerevisiae *gene required for cell wall integrity during budding and formation of mating projections

**DOI:** 10.1186/1471-2164-12-107

**Published:** 2011-02-14

**Authors:** Enrico Ragni, Heidi Piberger, Christine Neupert, Jesús García-Cantalejo, Laura Popolo, Javier Arroyo, Markus Aebi, Sabine Strahl

**Affiliations:** 1 University of Heidelberg, Centre for Organismal Studies (COS) Heidelberg, Cell Chemistry, Im Neuenheimer Feld 360, D-69120 Heidelberg, Germany; 2 Università degli Studi di Milano, Dipartimento di Scienze Biomolecolari e Biotecnologie, Via Celoria 26, 20133 Milan, Italy; 3 ETH Zürich, Institut für Mikrobiologie, Wolfgang-Pauli-Strasse 10, 8093 Zürich, Switzerland; 4 Unidad de Genómica-Campus Moncloa, Parque Científico de Madrid, Facultad CC. Biológicas, Universidad Complutense de Madrid, 28040 Madrid, Spain; 5 Departamento de Microbiología II, Facultad de Farmacia, Universidad Complutense de Madrid, 28040 Madrid, Spain

## Abstract

**Background:**

Mannoproteins construct the outer cover of the fungal cell wall. The covalently linked cell wall protein Ccw12p is an abundant mannoprotein. It is considered as crucial structural cell wall component since in baker's yeast the lack of *CCW12 *results in severe cell wall damage and reduced mating efficiency.

**Results:**

In order to explore the function of *CCW12*, we performed a Synthetic Genetic Analysis (SGA) and identified genes that are essential in the absence of *CCW12*. The resulting interaction network identified 21 genes involved in cell wall integrity, chitin synthesis, cell polarity, vesicular transport and endocytosis. Among those are *PFD1*, *WHI3*, *SRN2*, *PAC10*, *FEN1 *and *YDR417C*, which have not been related to cell wall integrity before. We correlated our results with genetic interaction networks of genes involved in glucan and chitin synthesis. A core of genes essential to maintain cell integrity in response to cell wall stress was identified. In addition, we performed a large-scale transcriptional analysis and compared the transcriptional changes observed in mutant *ccw12*Δ with transcriptomes from studies investigating responses to constitutive or acute cell wall damage. We identified a set of genes that are highly induced in the majority of the mutants/conditions and are directly related to the cell wall integrity pathway and cell wall compensatory responses. Among those are *BCK1*, *CHS3*, *EDE1*, *PFD1*, *SLT2 *and *SLA1 *that were also identified in the SGA. In contrast, a specific feature of mutant *ccw12*Δ is the transcriptional repression of genes involved in mating. Physiological experiments substantiate this finding. Further, we demonstrate that Ccw12p is present at the cell periphery and highly concentrated at the presumptive budding site, around the bud, at the septum and at the tip of the mating projection.

**Conclusions:**

The combination of high throughput screenings, phenotypic analyses and localization studies provides new insight into the function of Ccw12p. A compensatory response, culminating in cell wall remodelling and transport/recycling pathways is required to buffer the loss of *CCW12*. Moreover, the enrichment of Ccw12p in bud, septum and mating projection is consistent with a role of Ccw12p in preserving cell wall integrity at sites of active growth.

The microarray data produced in this analysis have been submitted to NCBI GEO database and GSE22649 record was assigned.

## Background

The fungal cell wall is a dynamic structure that acts as a permeability barrier for large molecules and is responsible for osmotic stability and cell shape. In *Saccharomyces cerevisiae*, the cell wall adds up to 30% of the total cell dry weight. It is mainly composed of polysaccharides (85%) and highly glycosylated mannoproteins (15%) [1]. The vegetative cell wall has a layered ultrastructure with an inner layer of β-1,3- and β-1,6-glucan and chitin, and an outer layer of mannoproteins. β-1,3-glucan is synthesized at the plasma membrane and constitutes the principal cell wall polysaccharide to which other components are cross-linked [2,3]. Chitin is synthesized by plasma membrane chitin synthases (*CHS1-3*) [4]. It is abundant at the bud neck and the primary septum between mother and daughter cells. In addition, chitin is a minor component of the lateral cell wall. Most of the chitin at the bud neck is linked to non-reducing ends of β-1,3-glucan, whereas the chitin of the lateral cell wall is mainly attached to β-1,6-glucan [4,5]. Soluble cell wall proteins (SCWs) are non-covalently associated with the carbohydrate meshwork [6]. In addition, Pir proteins (proteins with internal repeats; PIR-CWPs) are covalently linked to β-1,3-glucan, most likely via ester linkages between the γ-carboxyl group of glutamate residues, arising from glutamine, and glucose hydroxyl groups [7,8]. Moreover, at the outer layer of the β-1,3-glucan meshwork highly branched β-1,6-glucan is present to which mannoproteins are linked via a remnant of their glycosylphosphatidylinositol (GPI) anchor (GPI-anchored covalently linked cell wall proteins; GPI-CWPs) [4].

GPI-CWPs are either structural proteins or enzymes involved in cell wall biogenesis. They include the flocculins, the agglutinins, the *CRH *family, the *TIR *family, the *SED1-SPI1 *and *GAS *families [9,10]. Interestingly, the GPI-CWP Ccw12p cannot be assigned to one of these families. Loss of Ccw12p results in reduced growth rate and increased sensitivity to the cell wall perturbing agents Calcofluor White (CW) and Congo Red (CR), suggesting that Ccw12p is required for maintenance of cell wall stability [7,11]. In accordance, the amount of cell wall chitin is significantly increased in *ccw12*Δ cells, a feature of mutants with defects in cell wall integrity [12]. Electron microscopy of *ccw12*Δ cells revealed random deposition of cell wall material [11]. Most likely, the additional cell wall substance is reinforcing the inner glucan-chitin layer. Further, mutant *ccw12*Δ is especially sensitive to the aminoglycoside antibiotic hygromycin B, probably due to enhanced cell wall permeability [13]. Very similar phenotypes have been described for mutants that lack other GPI-CWPs involved in cell wall synthesis and organization. For example, the absence of Crh1p and Crh2p, which are required for the cross-linking of chitin to glucan, induces hypersensitivity to CR and CW [14].

Global transcriptional changes in response to deletion of genes crucial for cell wall biogenesis and remodelling (such as *GAS1*, *FKS1*, *KRE6 *and *MNN9*), exposure to cell wall perturbing agents (e.g., Zymolyase, CW and CR) and hyper-activation of the protein kinase C (PKC1)-dependent Cell Wall Integrity (CWI) pathway have been characterized [15-18]. These analyses revealed a major role for the CWI pathway in activating a compensatory mechanism to counteract different cell wall stress conditions and to prevent cell lysis [15,16,19]. The CWI pathway is activated by the plasma membrane sensor proteins Wsc1-3p, Mid2p and Mtl1p [20,21]. In response to wall perturbations the sensors stimulate exchange activity of the guanine nucleotide exchange factor Rom2p that is recruited to the plasma membrane via phosphatidylinositol 4,5-bisphosphate (PI4,5P2) [22]. Rom2p activates the small GTPase Rho1p that in turn activates among others Pkc1p [23]. Pkc1p turns on a mitogen-activated protein (MAP) kinase cascade consisting of the MAP kinase kinase kinase Bck1p/Slk1p, a pair of redundant MAP kinase kinases (Mkk1p and Mkk2p), and the MAP kinase Mpk1p/Slt2p. Slt2p phosphorylation triggers a number of cellular responses, one of which is the transcriptional activation of a variety of genes that have been implicated in cell wall assembly and structure [23]. Notably, genes involved in chitin biogenesis and cross-linking, *GFA1*, *CHS3 *and *CRH1*, were found to be up-regulated in many cell wall mutants [15]. Compensatory chitin deposition was demonstrated to be a crucial trait during cell wall damages and entirely dependent on the activity of Chs3p [24] and on the proteins controlling its transport and activation, Chs4-7p [25,26].

To learn more about the role of Ccw12p, we determined its sub-cellular localization and performed Synthetic Genetic Analysis (SGA) for mutations leading to growth defects when combined with a *CCW12 *deletion. In addition, we analysed global transcriptional changes in response to deletion of *CCW12*. Our findings suggest that both, Ccw12p and the CWI pathway are necessary to ensure cell wall integrity, especially during bud emergence and formation of the mating projection.

## Results

### Synthetic genetic interactions detected by mutant screening in a *ccw12*Δ background

Our previous electron microscopic studies and biochemical analyses of the cell wall of *ccw12*Δ mutants suggested a crucial structural role for Ccw12p during vegetative cell growth [11,12]. To gain further insight into the role of Ccw12p, we screened the Euroscarf collection of yeast mutants for mutations causing a growth defect when combined with a *CCW12 *deletion.

Haploid deletion mutants in 4869 genes marked by the resistance locus for G418 were robotically arrayed and crossed with a *ccw12::nat*^*R *^strain resistant to nourseothricin. The resulting diploid cells were selected, sporulated, and haploid double mutants scored for growth on medium containing both G418 and nourseothricin (see Methods for details). This selection procedure was performed twice to minimize technical errors and false positives. Double mutants showing no growth or very slow growth phenotypes were selected. We identified 129 mutants with putative genetic interactions with *ccw12*Δ. To verify these interactions, all identified mutant strains were sporulated, double mutant spores were selected and analysed for a synthetic phenotype. In this way, 21 *bona fide *synthetic interactions of *ccw12*Δ were confirmed. Nine of these double mutants did not grow (synthetic lethal = sl) whereas 12 mutants displayed very slow growth phenotypes (synthetic sick = ss; doubling times >10 hrs) (Table 1). Interestingly, 10 of the identified genes (*BCK1*, *BRE5*, *CHS3*, *CHS5*, *CHS7*, *EDE1*, *MID2*, *SHE4*, *SLA1 *and *SLT2*) have been described previously as *CCW12 *genetic interactors validating the quality of our screening procedure [27-31]. Moreover, the identification of 11 new interactors underlines the sensitivity of the screening. The 21 candidate genes were arranged into functional categories according to the Saccharomyces Genome Database (SGD):

**Table 1:** Genes showing synthetic interaction with *CCW12*

ORF	Gene	Description of gene product	SGA	**Fold change**^**(a)**^		**Interaction in other cell wall networks**^**(b)**^	**Published interaction **^**(c)**^
				Array	qRT-PCR		
		**Cell wall synthesis and regulation**					
*YBL061C*	*CHS4*	Activator of Chs3p (chitin synthase III), recruits Chs3p to the bud neck via interaction with Bni4p	ss			*FKS1, SMI1, GAS1*	-
*YBR023C*	*CHS3*	Chitin synthase III	ss	2.5	3.2 ± 0.7	*FKS1, SMI1, GAS1*	[27,30]
*YDR245W*	*MNN10*	Subunit of the Mannan polymerase II complex	sl			*CHS3, CHS4, CHS5, CHS6, CHS7, SMI1*	-
*YHR030C*	*SLT2*	MAP-kinase of the cell integrity pathway	sl	2.2	1.7 ± 0.1	*BNI4, CHS3, CHS4, CHS5, CHS6, CHS7, FKS1, GAS1, SMI1*	[31]
*YHR142W*	*CHS7*	Involved in chitin biosynthesis by regulating Chs3p export from the ER	ss			*FKS1, SMI1, GAS1*	[27,30,31]
*YJL095W*	*BCK1*	MAPKK-kinase of the cell integrity pathway	sl	2.5	2.7 ± 0.5	*BNI4, CHS1, CHS3, CHS4, CHS5, CHS7, GAS1, SMI1*	[31]
*YLR113W*	*HOG1*	Mitogen-activated protein kinase involved in osmoregulation	ss				-
*YLR330W*	*CHS5*	Involved in export of selected proteins, such as chitin synthase Chs3p, from the Golgi to the plasma membrane	sl			*FKS1, SMI1, GAS1*	[27,30]
*YLR332W*	*MID2*	Sensor for the PKC1-SLT2 cell wall integrity pathway	ss			*FKS1, SMI1*	[27,29,31]
		**Cell polarity and vesicular transport**					
*YBL007C*	*SLA1*	Cortical actin patch assembly control protein, mutation affects endocytosis	sl	2.7	2.7 ± 0.1	*CHS3, CHS4, CHS5, CHS7, FKS1, SMI1*	[31]
*YBL047C*	*EDE1*	Cortical actin patch protein with a role in endocytosis	ss	2.6	2.7 ± 0.5	*BNI4, CHS3, CHS4, CHS5, CHS7, FKS1*	[31]
*YCR034W*	*FEN1*	Involved in fatty acids elongation	ss			*FKS1*	-
*YDR129C*	*SAC6*	Involved in the organization and maintenance of the actin cytoskeleton	sl			*CHS3, CHS4, CHS5, CHS7*	-
*YJL179W*	*PFD1*	Involved in the biogenesis of actin and of alpha- and gamma-tubulin	sl	2.5	1.7 ± 0.4		-
*YLR370C*	*ARC18*	Required for the motility and integrity of cortical actin patches	sl			*CHS1, CHS3, CHS4, CHS5, CHS6*	-
*YNR051C*	*BRE5*	Activator of Ubp3p that regulates COPII coat assembly	ss			*BNI4, CHS3, CHS4, FKS1, SMI1*	[31]
*YOR035C*	*SHE4*	Involved in cortical actin patch assembly and endocytosis	ss			*CHS3, CHS4, CHS7, FKS1*	[31]
		**Other genes**					
*YLR119W*	*SRN2*	Component of the ESCRT-I complex, which is involved in ubiquitin-dependent sorting of proteins into the endosome	ss				-
*YNL197C*	*WHI3*	Regulates the critical cell size required for passage through Start	ss				-
*YDR417C*		Hypothetical protein, potential GPI anchor and secretion sequence	ss				-
*YGR078C*	*PAC10*	Polypeptide 3 of a Yeast Non-native Actin Binding Complex	sl				-

(a) Data correspond to the ratio of expression of *ccw12 *versus wt calculated as described in "Methods".

(b) [30,60]

(c) Published Genetic Interactions with *CCW12*.

#### Cell wall synthesis and regulation

Three of the identified genes are elements of the CWI pathway: the upstream sensor *MID2 *(ss), the MAP kinase kinase kinase *BCK1 *(sl) and the MAP kinase *SLT2 *(sl). Moreover, downstream targets of CWI pathway involved in chitin deposition were found: *CHS3 *(ss), a N-acetylglucosamine transferase responsible for chitin synthesis at the bud neck and in the lateral cell wall as well as for chitin deposition in response to cell wall stress; *CHS4 *(ss), a direct activator of Chs3p activity [32] anchoring Chs3p to the septin ring via Bni4p [33]; *CHS5 *(sl), a component of the exomer complex involved in the polarized transport of Chs3p in specialized vesicles [34]; and *CHS7 *(ss), a membrane protein controlling Chs3p export from the ER [35-37]. As a compensatory response, increased accumulation of chitin has been observed in *ccw12*Δ cells [12]. Moreover, deletion of chitin biosynthesis genes in a *ccw12*Δ background causes lethality, further proving the essential interconnection of Ccw12p and chitin in cell wall stability. Furthermore, we identified another target of the CWI pathway: *MNN10 *(sl), a Golgi mannosyltransferase involved in processing of N-linked glycans and septum formation [38,39]. Interestingly, simultaneous deletion of *HOG1 *and *CCW12 *resulted in a severe growth defect (ss). This result is consistent with previous findings identifying Hog1p, a MAP kinase involved in osmoregulation, as modulator of the response to cell wall damage [15-17,40,41].

#### Cell polarity and vesicular transport

Three genes required for endocytosis and polarization of the secretory pathway, *EDE1 *(ss) (key endocytic protein involved in a network of interactions with other endocytic proteins), *SHE4 *(ss) (regulator of myosin function), and *SLA1 *(sl) show severe synthetic phenotypes in combination with *ccw12*Δ. *SLA1 *is crucial for NPFXD-mediated endocytosis that is required for the function of the CWI pathway sensor Wsc1p, thus connecting endocytosis and cell wall integrity [42]. In addition, five genes involved in cell polarity caused a synthetic phenotype: *ARC18 *(sl) (motility and integrity of cortical actin patches), *SAC6 *(sl) (organization and maintenance of the actin cytoskeleton), *PFD1 *(sl) (biogenesis of actin and alpha- and gamma-tubulin), *BRE5 *(ss) (vesicular transport between the endoplasmic reticulum and Golgi compartments) and *FEN1 *(ss) (membrane biogenesis).

#### Other genes

Finally, genes belonging to other functional categories were found: *WHI3 *(ss), a cell size regulator required for passage through START; *SRN2 *(ss), a component of the endosomal sorting complex required for transport-I (ESCRT-I) which is involved in ubiquitin-dependent sorting of proteins into the endosome; *PAC10 *(sl), a co-chaperone promoting efficient protein folding, and *YDR417C *(ss), a gene of unknown function potentially coding for a GPI anchored protein.

### Phenotypic analysis of individual single mutants in genes identified by SGA

**Figure 1:**
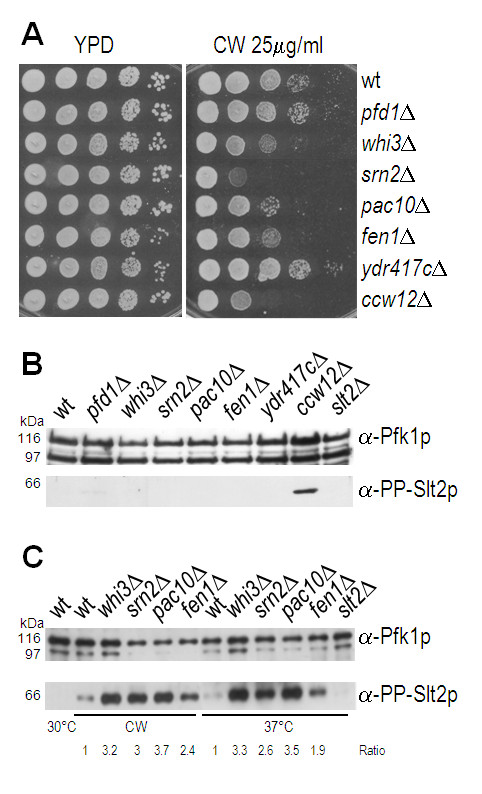
**Characterization of individual single mutants in genes identified by SGA**. **(A) **Calcofluor white (CW) sensitivity. Indicated mutant strains were processed as described in *Methods*. 3 × 10^5 ^cells, and 10-fold serial dilutions thereof were spotted on YPD plates with, and without 25 μg/ml of CW. Plates were incubated at 30°C for 2 days. **(B **and **C) **Activation of the CWI pathway. Cell extracts were analysed by Western blot using phospho-p44/p42 MAPK antibodies to detect the dually phosphorylated form of Slt2p, the MAP kinase of the CWI pathway (lower panel, PP-Slt2p). Phosphofructokinase 1 (Pfk1p) was used as a loading control (upper panel, Pfk1p antibodies are directed against both Pfk1p subunits). **(B) **To analyse constitutive Slt2p phosphorylation, wt and indicated mutant strains were grown at 30°C. **(C) **To analyse induction of Slt2p phosphorylation in response to different stresses, cells were incubated with CW or at elevated temperatures as described in Methods. For unstressed cells, one representative extract is shown. PP-Slt2p and Pfk1p protein levels were quantified using the ScionImage™ software, and the amount of PP-Slt2p was normalized to the amount of the larger Pfk1p subunit. Values of the mutants were referred to the wt value (set to 1), and are shown below the figure.

On the basis of computational analyses of large scale experiments *EDE1*, *SHE4*, *SLA1 *and *SAC6 *are predicted to be involved in cell wall organization, whereas *PFD1*, *WHI3*, *SRN2*, *PAC10*, *FEN1 *and *YDR417C *are not linked to cell wall synthesis, signalling or compensating mechanisms [43]. To further characterize the latter genes, we assayed CW sensitivity of the corresponding deletion mutant strains. CW interferes with the assembly of compensatory cell wall chitin. Therefore, CW sensitivity is a common feature of mutants with cell wall defects. Cells were spotted on solid rich medium containing 25 μg/ml CW, and incubated for 2 days at 30°C (Figure 1A). Due to the previously described phenotype [11], the *ccw12*Δ mutant was included as a control for growth inhibition. Growth of *pfd1*Δ and *ydr417c*Δ was not affected by CW (Figure 1A). In contrast, *pac10*Δ, *whi3*Δ and *fen1*Δ cells displayed intermediate, and *srn2*Δ cells high CW sensitivity.

Moreover, we analysed the CWI pathway in the CW sensitive deletion mutants, *pac10*Δ, *whi3*Δ, *fen1*Δ and *srn2*Δ, since constitutive activation of the CWI pathway is a characteristic phenotypic trait of mutants that directly affect cell wall structure. We addressed CWI pathway induction by measuring phosphorylation of the MAP kinase Slt2p. Slt2p activity increases as a result of tyrosine/threonine phosphorylation of the protein. Protein extracts were analysed by immunoblotting using α-phospho-p44/p42 MAP kinase antibodies, which detect the dually-phosphorylated form of Slt2p. The MAP kinase Slt2p was not activated in wild-type (wt) and *pac10*Δ, *whi3*Δ, *fen1*Δ and *srn2*Δ mutant strains under normal growth conditions (Figure 1B). However, cell wall stress conditions (CW treatment and increased temperature) resulted in hyper-activation of Slt2p in the mutants in comparison to wt cells (Figure 1C). Taken together, these results suggest that Pac10p, Srn2p, Whi3p and Fen1p are not directly involved in cell wall assembly but rather affect cell wall stress response. These proteins are involved in crucial processes such as protein folding, sorting into endosomes, cell cycle regulation and membrane lipid synthesis (Table 1). The corresponding mutants display reduced fitness with respect to various stress conditions (e.g., growth at 41°C; hygromycin B, ethanol treatment; http://www.yeastgenome.org/). Thus, our results suggest that in *pac10*Δ, *whi3*Δ, *fen1*Δ and *srn2*Δ cells hyper-activation of the CWI pathway in response to cell wall stress conditions is an indirect effect, although a direct regulation cannot be ruled out completely.

### Global transcriptional responses of the *ccw12*Δ mutant

In order to comprehensively assess the effect of the cell wall damage caused by *CCW12 *depletion, we examined the *ccw12*Δ transcriptome using DNA microarray technology, as described in Methods. Yeast strains were grown to an OD_600 _of 1 and total RNA was used to analyse expression levels of all *S. cerevisiae *genes present on the Affymetrix GeneChip^® ^Yeast Genome S98 array (Additional file 1).

Deletion of *CCW12 *resulted in transcriptional changes (fold change ≥2 or ≤ 0.5) of 473 genes (8% of the yeast genome) (Additional file 2). Interestingly, 91% of these genes were up-regulated, while only 9% showed reduced expression. Among the 431 up-regulated genes, 94% had 2 to 3-fold increased transcript levels, and 6% were more than 4-fold enhanced. In contrast, 41 genes were down-regulated, with 85% showing a 2 to 3-fold transcriptional reduction and 15% being reduced more than 4-fold. Transcriptome data were verified for selected genes using quantitative RT-PCR (qRT-PCR) (Table 1, Table 2 and Table 3) and Northern blot analysis (Additional file 3). Conformity between microarray, qRT-PCR and Northern blot data was obtained for the genes analysed. In the case of *ECM13*, transcript levels detected by qRT-PCR were less marked, possibly due to different gene regions experimentally verified. However, all experimental approaches point out significant induction of *ECM13 *transcription in *ccw12*Δ (Table 2 and Additional file 3).

**Table 2:** Cell wall related genes modulated in the *ccw12*Δ transcriptional response

ORF	Gene	Description of gene product	**Fold change**^**(a)**^	
			**Array**	**qRT-PCR**
*YBL001c*	*ECM15*	Cell wall biogenesis and architecture	2.2	
*YBL043w*	*ECM13*	Cell wall biogenesis and architecture	11.7	3.0 ± 0.9
*YBL101c*	*ECM21*	Cell wall biogenesis and architecture	2.4	
*YBR005w*	*RCR1*	Protein of the ER membrane involved in cell wall chitin deposition	2.3	
*YBR023c*	*CHS3*	Chitin synthase III	2.4	3.2 ± 0.7
*YBR038w*	*CHS2*	Chitin synthase II	2.3	
*YBR176w*	*ECM31*	Cell wall biogenesis and architecture	2.5	
*YBR199w*	*KTR4*	Putative mannosyltransferase	2.7	
*YBR205w*	*KTR3*	alpha-1,2-mannosyltransferase	2.2	
*YBR229c*	*ROT2*	Modification of N-linked oligosaccharides	2.3	
*YER011w*	*TIR1*	Cell wall mannoprotein	0.4	
*YGL038c*	*OCH1*	Modification of N-linked oligosaccharides	2.7	
*YGL259w*	*YPS5*	Protein with similarity to GPI-anchored aspartic proteases	4.4	2.6 ± 0.1
*YHR030c*	*SLT2*	Serine/threonine MAP kinase	2.2	1.7 ± 0.1
*YIL113w*	*SDP1*	Dual-specificity MAP kinase phosphatase	4.4	
*YIR019c*	*MUC1*	GPI-anchored cell surface glycoprotein (flocculin)	0.4	
*YIR039c*	*YPS6*	Putative GPI-anchored aspartic protease	4.4	
*YJL095w*	*BCK1*	Mitogen-activated protein (MAP) kinase kinase kinase	2.5	2.7 ± 0.5
*YJL099w*	*CHS6*	Mediate export of Chs3p from Golgi to PM	2.9	
*YJL128c*	*PBS2*	MAP kinase kinase of the HOG pathway	3.7	
*YJL139c*	*YUR1*	Mannosyltransferase of the KTR1 family	2.4	
*YJL174w*	*KRE9*	beta-1,6 glucan assembly	2.5	
*YJR131w*	*MNS1*	Alpha-1,2-mannosidase involved in N-linked glycosylation	2.1	
*YJR137c*	*ECM17*	Cell wall biogenesis and architecture	3.2	
*YKL161c*	*MLP1*	Putative ser/thr kinase with similarity to Slt2p	4.4	3.5 ± 0.3
*YKL163w*	*PIR3*	Cell wall mannoprotein	3.3	
*YKR076w*	*ECM4*	Cell wall biogenesis and architecture	3.4	
*YLR121c*	*YPS3*	GPI-anchored aspartic protease 3	2.9	
*YLR194c*	*YLR194C*	GPI-anchored cell wall protein	3.2	
*YOL007c*	*CSI2*	Structural component of the chitin synthase III complex	2.3	
*YOR382w*	*FIT2*	GPI-anchored cell wall protein	3.3	
*YPL163c*	*SVS1*	Cell wall and vacuolar protein	2.3	

(a) Data correspond to the ratio of expression of *ccw12 *versus wt calculated as described in "Methods".

**Table 3:** Mating related genes modulated in the *ccw12*Δ transcriptional response

ORF	Gene	Description of gene product	**Fold change**^**(a)**^	
			**Array**	**qRT-PCR**
*YBR040w*	*FIG1*	Integral membrane protein required for efficient mating	0.4	0.3 ± 0.1
*YCL027w*	*FUS1*	Membrane protein localized to the shmoo tip, required for cell fusion	0.5	0.5 ± 0.1
*YCR089w*	*FIG2*	Cell wall adhesin, expressed specifically during mating	0.4	
*YDL039c*	*PRM7*	Pheromone-regulated protein	0.4	
*YGL089c*	*MF(ALPHA)2*	Mating pheromone alpha-factor	0.4	
*YIL117c*	*PRM5*	Pheromone-regulated protein induced during cell integrity signalling	2.8	
*YJL108c*	*PRM10*	Pheromone-regulated protein	3.7	2.4 ± 0.2
*YML047c*	*PRM6*	Pheromone-regulated protein	0.5	
*YNL279w*	*PRM1*	Pheromone-regulated multispanning membrane protein	0.4	
*YNR044w*	*AGA1*	Anchorage subunit of a-agglutinin of a-cells	0.4	
*YPL156c*	*PRM4*	Pheromone-regulated protein	0.4	
*YPL192c*	*PRM3*	Pheromone-regulated protein	0.3	0.7 ± 0.2

(a) Data correspond to the ratio of expression of *ccw12 *versus wt calculated as described in "Methods".

**Figure 2:**
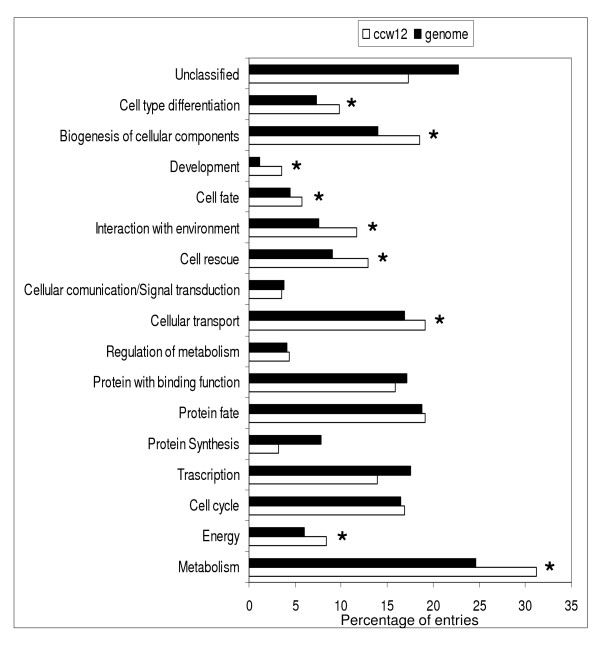
**Functional categories of the differentially expressed genes**. *Black *bars represent the functional catalogue of 6200 genes according to the MIPS classification. *White *bars represent the distribution of the 472 differentially expressed genes from *ccw12*Δ mutant according to the MIPS functional catalogue. The percentage of entries is given by the ratio of the number of regulated genes in each category and the total number of differentially expressed genes. Asterisks highlight categories with p-values < 0.05. P-values were obtained using hypergeometric distribution statistics (default setting in FunCatDB tools, MIPS). Note that the sum of entries is more than 100% since some genes are annotated in more than one functional category in the FunCat database.

To further analyse the transcriptional profile of the *ccw12*Δ mutant, differentially expressed genes were grouped into functional categories according to the MIPS nomenclature (http://mips.helmholtz-muenchen.de). We expressed the number of up- and down-regulated transcripts in each of the categories as percentage of the total number of differentially expressed genes and compared this functional classification with the functional catalogues of genes described in MIPS. As a result the major remodelling of gene expression is accentuated on a genomic scale (Figure 2). Transcripts encoding products involved in central metabolism, generation of energy, cell rescue and biogenesis of cellular components are significantly overrepresented in mutant *ccw12*Δ (p-value < 0.05; p-values were obtained using hypergeometric distribution statistics; default setting in FunCatDB tools, MIPS). In contrast, transcripts encoding products involved in protein synthesis and transcription were underrepresented (although with a p-value > 0.05), consistent with the fact that cell proliferation is impaired by mutations affecting cell wall biogenesis [44]. At present it cannot be completely excluded that part of the observed responses, especially related to transcription and protein biosynthesis, are due to the reduced growth rate of the *ccw12*Δ mutant (approximately 25% less than wt).

Among the differentially expressed genes, 7% (32 out of 472 genes) are directly involved in the construction and remodelling of the cell wall (Table 2). Most of these genes showed up-regulation while only two, *MUC1 *and *TIR1*, have reduced expression. These findings indicate a strong bias towards remodelling of the cell wall in the *ccw12*Δ mutant to compensate for defects. Furthermore, the DNA microarray data revealed that some of the characteristic target genes of the CWI and HOG pathways [45,18,46] were affected in the *ccw12*Δ mutant. As shown in Table 2, we found regulation of *BCK1*, coding for the MAPKKK of the pathway; *SLT2*, the terminal kinase; *MLP1*, encoding a putative Ser/Thr kinase with similarity to Slt2p; and *SDP1*, whose product is a dual-specificity MAP kinase phosphatase acting on and regulating the phosphorylated/active form of Slt2p [23]. *SLT2 *levels increased about 2.2 times, in agreement with a similar modulation reported for other cell wall mutants such as *kre6*Δ, *gas1*Δ or *mnn9*Δ [15]. Downstream targets of the CWI pathway included genes involved in chitin synthesis (*RCR1*, *CSI2, CHS2*, *CHS3 *and *CHS6*) [47] and β-1,6-glucan synthesis (*KRE9*) [48] as well as mannoprotein-encoding genes, such as Pir3p, the putative GPI-anchored Fit2p and Ylr194cp, and GPI-anchored aspartic proteases (yapsines *YPS3*, *YPS5 *and *YPS6)*, whose role in the cell wall construction has been inferred from genetic studies [49]. Further, we found transcriptional induction of *PBS2*, the MAPKK gene of the HOG pathway.

Another group of up-regulated genes is involved in the biosynthesis of protein-linked glycans, including *MNS1*, which encodes the ER mannosidase I, and *ROT2*, encoding a luminal α-glucosidase II of the ER. Mns1p and Rot2p are involved in the trimming of N-linked glycans in the ER [50,51]. Similarly, up-regulation was found for *OCH1 *and *YUR1*, coding for Golgi α-1,6- and α-1,2-mannosyltransferases, respectively, both being involved in the maturation of N-linked oligosaccharides [51]; and for *KTR3 *and *KTR4*, encoding α-1,2-mannosyltransferases involved in the outer chain synthesis of the N-glycans and in the elongation of O-linked oligosaccharides [51].

Beside the alterations in the expression levels of glycosyltransferases and glycosidases, up-regulation of *ECM4-13-15-17-21-31 *was observed. These genes encode proteins that are poorly characterized or have unknown functions. However, due to the corresponding mutant phenotypes these genes are thought to play a role in different aspects of cell wall construction.

### Comparison of the *ccw12*Δ transcriptome with transcriptional profiles from other cell wall-related mutants

**Figure 3:**
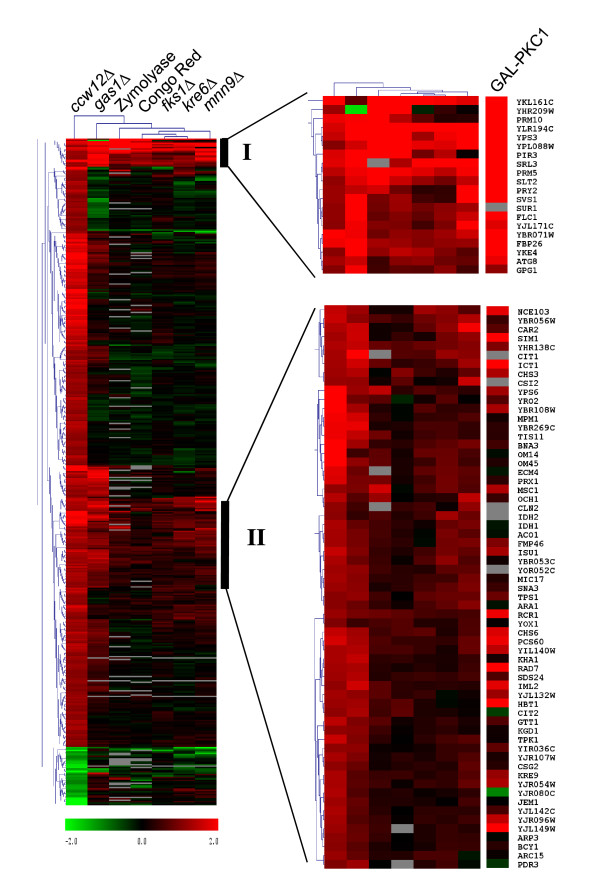
**Hierarchical clustering of genes differentially expressed in *ccw12*Δ**. The transcriptional response of the *ccw12*Δ mutant was compared to transcriptomes of other cell wall mutants (*gas1*Δ, *fks1*Δ, *mnn9*Δ and *kre6*Δ) and cell wall stress conditions (Zymolyase and Congo Red) [15,16]. Each column represents a different condition. Each row represents the ratio of expression for each gene as it is indicated in the colour scale. The clustering tree was built using MeV software from TIGR.

In order to compare transcriptional changes in the *ccw12*Δ mutant with transcriptomes from previous studies investigating responses to constitutive cell wall damage caused by deletions of cell wall-related genes, such as *GAS1*, *FKS1*, *KRE6 *and *MNN9*, and transient cell wall damage induced by CR or Zymolyase treatment [15,16], data were organized by hierarchical clustering (see Methods). As shown in Figure 3, two specific gene clusters could be identified.

Cluster I includes 20 genes that were highly induced in the majority of the mutants/conditions analysed. These genes are directly related to cell wall compensatory responses: the CWI pathway MAP kinase *SLT2 *and its homolog *MLP1 *(*YKL161c*); the known CWI pathway downstream targets *PRM5; *the PIR-CWP *PIR3*; and four genes coding for GPI-anchored proteins (*YLR194c*, *YPS3*, *SVS1 *and *YJL171c*). In addition, cluster I includes genes whose absence causes increased stress sensitivity: *FLC1*, coding for a putative flavin adenine dinucleotide (FAD) transporter required for uptake of FAD into endoplasmic reticulum and cell wall maintenance via disulphide bond formation [52]; *PRY2*, encoding a protein of unknown function (null mutant has a decreased resistance to hyperosmotic stress); *SUR1*, coding for a mannosylinositol phosphorylceramide (MIPC) synthase involved in sphingolipid biosynthesis (null mutant is sensitive to caffeine); *FBP26*, encoding a fructose-2,6-bisphosphatase (null mutant has a decreased resistance to hyperosmotic stress); and *YKE4*, coding for a zinc transporter (null mutant has decreased resistance to CW). Except for *SUR1*, all of these genes are activated in a strain with hyper-activated *PKC1 *[53], and thus can be considered as targets of the CWI pathway (Figure 3, right column). Accordingly, promoter analyses of these genes revealed the presence of one or more putative binding sites for the transcription factors of the CWI pathway, *RLM1 *and/or *SWI4 *(data not shown).

Cluster II contains genes which are moderately up-regulated under most of the analysed conditions (64 genes). Many of them are also induced upon *PKC1 *overexpression (Figure 3). This cluster includes functional categories like heat shock response and metabolism, comprising 8 genes connected to sugar, glycoside, polyol and carboxylate catabolism (*TPS1*, *ARA1*, *KGD1*, *YJR096W*, *ACO1*, *IDH1*, *CIT1 *and *IDH2*) and 6 genes involved in the tricarboxylic-acid pathway (*CIT2*, *KGD1*, *ACO1*, *IDH1*, *CIT1 *and *IDH2*).

**Figure 4:**
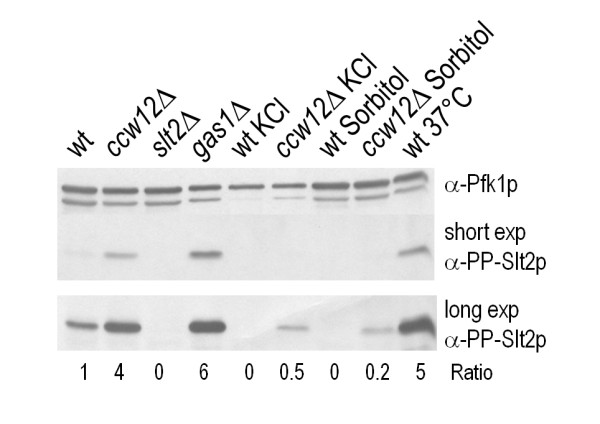
**Activation of the CWI pathway in mutant *ccw12*Δ**. Mutant *ccw12*Δ, *slt2*Δ, *gas1*Δ and BY4741 (isogenic wt) cells, were grown to mid-exponential phase, and cell extracts were analysed by Western blot using phospho-p44/p42 MAPK antibodies to detect dually phosphorylated Slt2p (mid and low panel, PP-Slt2p). Pfk1p was used as a loading control (upper panel, Pfk1p). PP-Slt2p and Pfk1p protein levels were quantified as described in Figure 1

To further verify the importance of the CWI pathway for *ccw12*Δ cells, we analysed the activation of Slt2p. As positive controls for Slt2p activation, we used cell extracts from *gas1*Δ cells and from wt cells incubated at 37°C for three hours. Compared to the wt, the *ccw12*Δ mutant displayed a 3.85-fold higher level of phosphorylated Slt2p (Figure 4). Addition of 1 M Sorbitol or 0.5 M KCl for osmotic stabilization greatly reduced the phosphorylation of Slt2p in *ccw12*Δ cells.

In summary, loss of *CCW12 *activates the CWI pathway which in turn up-regulates the expression of a distinct subset of genes that is also induced in all the other cell wall stress conditions analysed so far. Moreover, this pathway ensures cell viability of mutant *ccw12*Δ as emerged in the SGA screen.

### Ccw12p is crucial for cell wall stability of forming daughter cells

**Figure 5:**
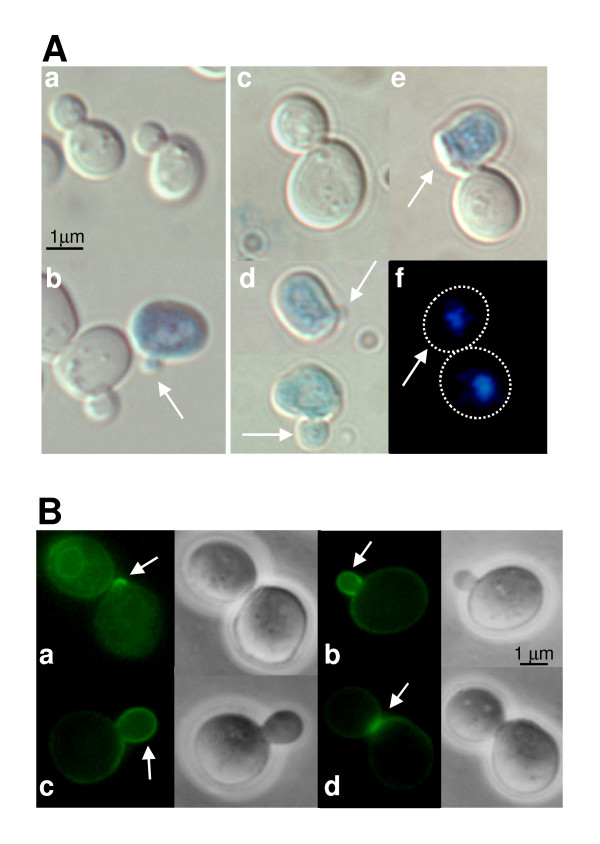
**Ccw12p localizes to areas of active cell wall synthesis and *ccw12*Δ cells display bud lysis**. **(A) **Cell lysis phenotypes. Wt (SEY6211) and *ccw12*Δ mutant (MEY12B) strains, exponentially growing in YPD **(a, b) **or YPD supplemented with 1 M sorbitol **(c, d)**, were stained with the vital dye methylene blue to identify dead cells (details are described in Methods): **(a) **wt cells display a typical ellipsoidal shape; **(b) **mutant cells show a pronounced round morphology and lyse as small budded cells (16% of *ccw12*Δ cells vs. 3% of wt cells); **(c) **mutant cells round morphology is partially reverted in presence of osmotic stabilization; **(d) **After hypotonic shock (transfer to YPD) lysis as small budded cell is observed(41% of *ccw12*Δ cells vs. 8% of wt cells) and **(e-f) **cell lysis occurs after completion of cytokinesis as shown by DAPI staining (panel e and f represent the same cells that have been stained with methylene blue and DAPI). **(B) **Localization of Ccw12p during vegetative growth. To exclude artefacts due to over-expression of CCW12-GFP, Ccw12p-GFP is expressed from plasmid pCCW12-GFP in mutant MEY12B. **(a) **Ccw12p-GFP is enriched at sites of emerging buds. The arrow marks the site of bud emergence. When cells proceed in the cell cycle, Ccw12p-GFP is specifically enriched in small **(b) **and medium-sized **(c) **buds. **(d) **After cytokinesis Ccw12p-GFP marks the septum.

During vegetative growth, the CWI pathway is essential for bud formation [23]. Since *ccw12*Δ mutants depend on the CWI pathway (see above) and produce buds that are abnormally round [54], we speculated that Ccw12p could play a role in maintaining cell wall integrity during bud growth. Thus, we analysed the mutant for abnormalities during vegetative growth. When grown in YPD medium, buds of *ccw12*Δ cells appeared rounder when compared to buds of wt cells. Bud morphology was further monitored by determining the relation between the length of the long and short cell axis. In contrast to wt cells (axes ratio of 1.2 ± 0.1), the long/short axis ratio was diminished in *ccw12*Δ cells (axes ratio 0.8 ± 0.1). Furthermore, 16% of *ccw12*Δ cells showed cell lysis during bud emergence as compared to only 3% of wt (Figure 5A, a and b). Addition of 1 M Sorbitol to the growth medium could partially prevent cell lysis (10%) and round bud morphology (axes ratio 1.1 ± 0.1) of *ccw12*Δ cells (Figure 5A, c). Moreover, when *ccw12*Δ cells were shifted from osmotically stabilized medium (YDP plus 1 M Sorbitol) to YPD (hypotonic shock), buds showed pronounced lysis. While 41% of the mutant cells underwent cell death mainly in early stages of bud formation, only 8% of wt cells were affected (Figure 5A, d). To a minor extent, cells lysed after completion of cytokinesis, but, in that case lysis was mainly restricted to daughter cells (Figure 5A, e and f).

To further study the role of Ccw12p in bud formation, we analysed its sub-cellular localization. For this purpose we constructed a plasmid encoding a Ccw12p-GFP fusion protein. The GFP-coding sequence was inserted into the *CCW12-HA *gene [11] at bp +129 without altering the N-terminal signal sequence and the GPI attachment site. Transcription of GFP fusion was under the control of the endogenous *CCW12 *promoter. The Ccw12p-GFP fusion protein was completely functional since it was able to complement all described mutant phenotypes, i.e. the slow growth phenotype (wt T_d _= 1.7 hrs; *ccw12*Δ T_d _= 2.2 hrs; *ccw12*Δ plus Ccw12p-GFP T_d _= 1.8 hrs) and the CW hyper-sensitivity (data not shown). Fluorescence microscopy revealed a uniform GFP signal around the cell surface confirming the presence of Ccw12p in the plasma membrane/cell wall. Interestingly, Ccw12p-GFP appeared more concentrated at the presumptive budding site on the cell surface of the mother cell shortly before bud emergence (Figure 5B, a). Concomitant with bud emergence, Ccw12p-GFP strongly accumulated at the periphery of small to medium-sized buds (Figure 5B, b and c). When buds reached a larger size, the protein appeared in the lateral cell wall of daughter cells and labelled the septum region with an asymmetric localization towards the daughter side. Concurrent with mother/daughter cell separation, Ccw12p-GFP was still detected on the daughter side of the cells (Figure 5B, d). Thereafter, the Ccw12p-GFP signal vanished, suggesting a specific function related to the stability of the growing cell wall and the septum. Interestingly, a similar distribution was found for upstream sensors and other components of the CWI pathway, further underlining the crucial role of monitoring correct assembly and structural capacity of the newly synthesized cell wall [55].

In summary, the cell lysis phenotype of *ccw12*Δ mutants and the localization of Ccw12p-GFP are supporting an essential function of Ccw12p for cell wall integrity predominantly during the formation of daughter cells.

### Ccw12p is involved in the mating process

The observed mating and agglutination defects of *ccw12*Δ mutants indicate a role of *CCW12 *in conjugation [7]. In accordance with these findings, our transcriptome analysis revealed significant regulation of genes involved in different steps of the mating process (Table 3) and hierarchical cluster analysis further demonstrated that this response is specific for *ccw12*Δ cells and not observed in other cell wall mutants (data not shown). The gene encoding the mating pheromone α-factor, *MF(ALPHA)2*, showed a 3-fold reduction and genes coding for cell wall or plasma membrane proteins involved in mating such as *FIG1*, *FIG2 *and *FUS1 *were down-regulated as well. Further, transcription of a set of pheromone-regulated genes (*PRM1-3-4-6-7*) required for different steps of membrane and nuclear envelope fusion was reduced. Surprisingly, the pheromone-regulated gene *PRM5 *was significantly activated (2.84-fold). However, *PRM5 *is induced by the CWI pathway [18] and thus activation by cell wall stress might overcome the repression during mating response. Finally, *PRM10*, that was proposed to be involved in mating, was up-regulated 3.67 fold, possibly due to the presence of one Rlm1p and two Swi4p binding sites in its promoter (data not shown).

**Figure 6:**
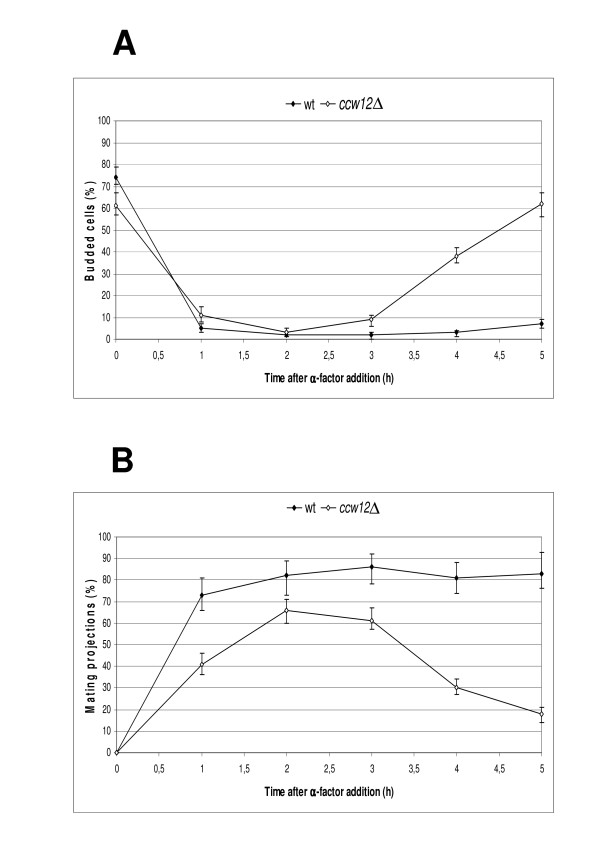
**Budding and formation of the mating projection are affected in *ccw12*Δ cells**. Wt (SEY6211) and *ccw12*Δ mutant (MEY12B) strains were treated with the mating pheromone α-factor as detailed in *Methods*. At time zero 20 μg/ml of α-factor were added. Budding index **(A) **and shmoo formation **(B) **was monitored over the time. Mean values of four independent experiments are shown. At least 200 cells were analysed at the indicated times.

To further substantiate the indicated role of Ccw12p during the mating process, we analysed the response of *ccw12*Δ cells to mating pheromone. During pheromone treatment, cells arrest in G1 and undergo polarized growth, forming a mating projection that is directed towards the signal source. This chemotropic response is thought to involve the generation of an internal landmark that reflects the direction of incidence of the external pheromone signal and overrides the spatial cues that normally control bud formation [56,57]. Although transcriptome analysis was performed in *ccw12*Δ *MAT***alpha **cells, we further characterized the pheromone response of *MAT***a **cells since the mating pheromone α-factor is commercially available, and for both, *ccw12*Δ *MAT***a **and *MAT***alpha **cells agglutination and conjugation defects have been demonstrated [7]. However, minor differences cannot be completely excluded. Wt and *ccw12*Δ *MAT***a **cells were treated with 20 μg/ml of α-factor. Budding index (Figure 6A) and formation of mating projections (shmoos) (Figure 6B) were monitored up to five hours after α-factor addition. In wt and *ccw12*Δ cells the percentage of budded cells decreased from 74% and 62%, respectively, to 2-3% after two hours of pheromone treatment. The percentage of budded wt cells remained constant with only a slight increase to 7% of budded cells after five hours (Figure 6A). Different from that, after 3 hours the number of budded *ccw12*Δ cells rapidly increased to reach 62% five hours after α-factor addition (Figure 6A) suggesting an earlier release from the G1 arrest.

**Figure 7:**
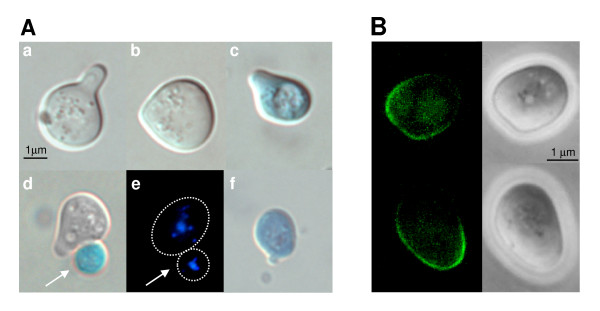
**Ccw12p localizes to the shmoo tip and *ccw12*Δ cells display pheromone induced cell lysis**. **(A) **Wt (SEY6211) and *ccw12*Δ mutant (MEY12B) strains were treated with α-factor (20 μg/ml) for two hours. Cells were stained with the vital dye methylene blue. **(a) **Wt cells display a typical elongated shmoo. **(b) **Shmoos of mutant cells are rounder and less polarized. **(c) **Mutant cells dye during shmoo formation (10% of *ccw12*Δ vs. 1% of wt cells). **(d, e) **Buds of mutants cells released from G1-arrest undergo cell lysis after cytokinesis is completed; a representative cell stained with methylene blue **(d) **and DAPI **(e) **is shown. **(f) **Mutant cells tend to lyse as small budded cells after re-entry of the mitotic cell cycle (23% of *ccw12*Δ vs. 5% of wt cells). **(B) **Localization of Ccw12p-GFP to the shmoo during pheromone treatment. Yeast strain described in figure 7B was treated with α-factor as indicated in 7A.

Mating projections of *ccw12*Δ cells were rounder and less polarized than those of wt cells (Figure 7A, a and b). Moreover, methylene-blue vital-staining showed that about 10% of the *ccw12*Δ cells undergo mating-induced cell death (versus 1% of wt cells) (Figure 7A, c). In addition, some shmoo-forming cells are still attached to dead daughter cells (Figure 7A, d-e). Further, 23% of *ccw12*Δ mutant cells that are exiting the pheromone block undergo cell lysis during bud emergence (versus 5% of wt cells) (Figure 7A, f). These data support a role for Ccw12p in stabilizing the cell wall during shmoo formation and during budding upon re-entry into the mitotic cell cycle.

The mating projection is a crucial structure during mating since it serves to concentrate the proteins involved in signalling (pheromones and pheromone receptors), cell adhesion (agglutinins) and fusion (Fus2p) in the area of future cell contact and fusion [58]. Interestingly, components of the CWI pathway, such as the cell wall integrity sensor Mid2p [59], accumulate in the same area suggesting a crucial role for cell wall construction and sensing during this process. Therefore, we used the GFP fusion to monitor Ccw12p localization during α-factor treatment. As shown in Figure 7B, Ccw12p-GFP is enriched in mating projections with a gradient starting from the tip of the shmoo. The same localization of Ccw12p-GFP was observed when shmoo formation was triggered in *MAT***alpha **cells by the presence of *MAT***a **cells (data not shown). In combination our data reinforce the notion that Ccw12p plays a general role in cell wall stability at the shmoo apex.

## Discussion

In this work we provide evidence that Ccw12p has a specific role in maintaining the stability of the newly synthesized cell wall especially during bud emergence and formation of the mating projection. Ccw12p localizes to areas of active cell wall synthesis such as forming buds (Figure 5B). In the absence of Ccw12p, buds are abnormally round [54] and cells tend to lyse during bud emergence and development (Figure 5A, panel b; 16% of *ccw12*Δ vs. 3% of wt cells). Moreover, when *ccw12*Δ cells are subjected to hypotonic shock buds lyse more frequently in early stages of development (Figure 5A, panel d; 41% of *ccw12*Δ cells vs. 8% of wt cells). Even after cytokinesis, daughter cells tend to lyse (Figure 5A, panel e). In agreement with the observed lysis phenotype, Ccw12p is specifically localized at the daughter side of the septum (Figure 5B, panel d). During mating, cell lysis is observed when the shmoo is forming (Figure 7A, panel c; 10% of *ccw12*Δ cells vs. 1% of wt cells) and Ccw12p is specifically localized to the mating projection (Figure 7B), further supporting a local function for cell wall stability during remodelling.

The CWI pathway plays a crucial role in cell wall stability especially during bud and shmoo formation. Mutants in this pathway show some of the phenotypic traits that are characteristic for *ccw12*Δ [23]. We could demonstrate, that in *ccw12*Δ cells the pathway is hyper-activated (Figure 4) and that osmotic stabilization suppresses CWI pathway hyper-activation (Figure 4) as well as the growth defect of *ccw12*Δ [12]. In addition, SGA and transcriptome analyses further demonstrate the interdependence between components of the CWI pathway and *CCW12 *(Tables 1 and 2). SGA revealed synthetic lethality of the *CCW12 *deletion in combination with mutants of the key regulatory kinases of the CWI pathway, *BCK1 *and *SLT2 *(Table 1). In addition, combinatory deletions with chitin synthesis genes (Table 1; [60]), and *MID2*, one of the main sensors of the CWI pathway, (Table 1; [29]) displayed severe synthetic growth phenotypes. Surprisingly, no direct involvement of the sensors *WSC1-3 *[23] was found. Wsc1p normally localizes to sites of new cell surface growth as similarly observed for Ccw12p-GFP during the budding process. However, we could demonstrate an interaction with *SLA1*. It is known that Sla1p, a component of the actin- and clathrin-based endocytic machinery can serve as an NPFX_(1,2)_D-specific endocytic adaptor and that the NPFX_(1,2)_D-Sla1p system is responsible for directing Wsc1p into an endocytic and recycling pathway necessary to maintain yeast cell wall polarity [42]. In turn, inhibition of Wsc1p endocytosis causes defects in polarized deposition of cell wall material and increased sensitivity to perturbation of cell wall synthesis. In our analysis, *SLA1 *deletion conferred synthetic lethality in combination with *ccw12*Δ (Table 1), similar to what was found in independent screens performed combining deletions of *SLA1 *and genes involved in glucan and chitin synthesis [60,30]. Moreover, the *SLA1 *transcript is 2.69-fold increased when *CCW12 *is absent (Table 1). These results suggest that NPFX_(1,2)_D/Sla1p-mediated endocytosis and polarized localization of Wsc1p are important to ensure cell wall integrity in the developing daughter cell in the absence of Ccw12p. The lack of synthetic lethality of *CCW12 *in combination with *WSC1 *could be explained by a redundant function of one of the other *WSC *sensors. In contrast, deletion of *SLA1 *might not only affect *WSC1 *but other WSC-family members as well.

Transcriptional profiling of the *ccw12*Δ mutant revealed predominantly cell wall remodelling activities. Members of all categories of cell wall related genes are regulated: CWI pathway signalling, structural components, chitin/glucan synthesis and glycosylation with many of them under the control of PKC1-mediated signalling cascade. Interestingly, despite the high transcriptional activation of some of these genes, their deletion mostly generates mild or no cell wall phenotypes and only a few of them conferred synthetic lethality when deleted in combination with *CCW12*. This may either be explained by functional redundancy or the need to repress several cell wall functions and/or components in order to cause severe cell wall damage.

In addition to the CWI pathway, the high-osmolarity glycerol (HOG) and the SLN1-SKN7 pathway contribute to cell wall integrity, The HOG pathway is modulated by various plasma membrane sensors [61]. One of which is the kinase Sln1p, the initiating member of a two-component type of phospho-relay cascade that negatively regulates the HOG pathway. In addition, Sln1p activates the transcription factor Skn7p which in turn triggers the transcription of specific target genes such as *OCH1 *and *NCA3 *[13]. CWI and HOG pathway are closely interconnected suggesting a cooperative role for both pathways controlling cell integrity [15-17,40,41]. Likewise, it was suggested that the CWI and the SLN1-SKN7 pathway may function in parallel to protect cells from lysis [13]. Interestingly, SGA revealed the interdependence between *CCW12 *and *HOG1*, the MAP kinase of the HOG pathway (Table 1). Further, transcription of *OCH1 *and *NCA3 *is significantly increased in the *ccw12*Δ mutant (2.69- and 3.36-fold increase respectively; Additional file 2) confirming previous data that suggested Ccw12p to be a modulator of Sln1p activity [13]. Thus, different signalling pathways are involved to ensure cell wall integrity in the absence of Ccw12p. However, the CWI pathway and cell wall remodelling genes play a dominant role as demonstrated by the preponderance of CWI related genes identified in the SGA screen.

We further addressed the question whether different cell wall defects induce a similar response in yeast cells. Although there is some variation in the regulation of individual genes under different conditions, their functional classification indicates a common principle. We found significant overlap in the transcriptional response to *CCW12 *deletion as compared to that in other cell wall mutants and treatment with cell wall perturbing agents such as Zymolyase or CR [15,16]. Notably, under all tested conditions we found 20 genes to be strongly induced, which could constitute the core response for cell wall stress (Figure 3, Cluster I). With the exception of *SUR1*, all the genes in this cluster are CWI pathway targets, reinforcing the notion that survival under cell wall stresses is mediated by different aspects regulated by CWI pathway.

In a previous work, a marked decrease in the mating and agglutination ability of *ccw12*Δ strain was detected [7]. Here we demonstrate that *ccw12*Δ cells exhibit diminished α-factor sensitivity and earlier release from the pheromone-induced G1 arrest (Figure 6). The mating-related phenotypes could be due to a combination of different factors: i) during pheromone treatment, the absence of Ccw12p at the shmoo apex results in mating induced cell death of ~10% of the cells (Figure 7A). As a result, mating frequency is significantly reduced. ii) The *ccw12*Δ expression profile revealed down-regulation of genes involved in the mating process which is not detected in other cell wall mutants. iii) *ccw12*Δ cells show an early release from the pheromone-induced G1-arrest. The time cells display mating projections and the recovery time from pheromone arrest can greatly influence the mating efficiency. It is well known that in the absence of a successful mating event, G1-arrested cells re-enter the mitotic cycle through a recovery process that involves down-regulation of the mating mitogen-activated protein kinase cascade mainly through Msg5p phosphatase activity on the MAP kinase, Fus3p [62]. Furthermore, recovery is regulated by *POG1*, coding for a putative transcriptional activator [63]. Overexpression of *POG1 *inhibits α-factor-induced G1-arrest and transcriptional repression of the *CLN1 *and *CLN2 *genes. This loss of transcriptional repression occurs through SCB/MCB promoter elements and requires Bck1p, known to up-regulate Swi4-dependent cell-cycle box (SCB)/*Mlu*I cell-cycle box (MCB) promoter elements during vegetative growth [64]. The G1-cyclin Cln2p, in addition to driving the G1- to S-phase transition [65], when over-expressed blocks the ability of cells to arrest in the presence of α-factor, primarily through an effect on Ste20p, an activator of the mating MAPK cascade [66]. In agreement with these data, we detected a 3.14-fold increase for *POG1 *and a 2.18-fold increase for *CLN2 *in *ccw12*Δ cells, explaining the earlier G1-release.

## Conclusions

Transcriptome and synthetic genetic analyses revealed that in *ccw12*Δ cells a cell wall compensatory mechanism is activated that integrates several major regulatory systems mainly under control of the CWI pathway-signalling module. Further, we demonstrate for the first time that Ccw12p is mainly localized in areas of newly synthesized cell wall such as emerging buds and mating projections. Our data strongly suggest that Ccw12p has a specific role in reinforcing the expanding cell wall. It will be a challenging future task to unravel the molecular mechanism of Ccw12p action.

## Methods

### Yeast strains and plasmids

Standard procedures were used for all DNA manipulations [67]. All cloning and transformations were made in *E. coli *strain DH5α. Oligonucleotide sequences are available upon request.

Plasmid pCCW12-GFP: a 741 bp DNA fragment corresponding to the coding sequence of *Aequorea victoria *GFP was amplified by PCR and subcloned into the *Bam*HI restriction site of pCCW12 [11]. Correct orientation of the insert was verified by restriction digestion and DNA sequencing.

*S. cerevisiae *strains used in this work are: SEY6210 (*MAT***α ***ura3-52 leu2-3,112 his3*-Δ*200 trp1*-Δ*901 lys2-801 suc2*-Δ *GAL *[68]); SEY6211 (*MAT***a ***leu2-3,112 ura3-52 his3-*Δ*200 trp1-*Δ*901 suc2-*Δ*9 GAL *[68]); MEY12A (SEY6210 except *ccw12::URA3 *[7]); MEY12B (SEY6211 except *ccw12::URA3 *[7]); BY4741 (*MAT***a ***his3*Δ*1 leu2*Δ*0 met15*Δ*0 ura3*Δ*0 *[69]); ΔarrayORF (BY4741 except *orf*Δ*::KanMX4 *[69]); Y3656 (*MAT***α ***can1*Δ*::MFA1pr-HIS3-MF*α*1pr-LEU2 his3*Δ*1 leu2*Δ *lys2*Δ *ura3*Δ [28]). ER*ccw12*Δ (Y3656 except *ccw12*Δ*::NatMX4*): To delete *CCW12 *in strain Y3656 we used the short-homology PCR technique, followed by one-step gene disruption [70]. Briefly, PCR primers complementary to nucleotides +1 to +60 and +343 to +402 of the *CCW12 *coding sequence were used to amplify the NatMX4 marker cassette conferring resistance to clonNAT (nourseothricin) from plasmid pAG25. The resulting PCR fragment was transformed into strain Y3656. Correct integration was verified by PCR on genomic DNA.

Yeast cells were grown in synthetic complete (SD) or YPD medium at 30°C. Haploid deletion mutants were available from the deletion project consortium EUROSCARF. These strains were arrayed in duplicate on fifty-one 384-format plates using a colony picker. Arrays were propagated at 30°C on YPD or YPD supplemented with 200 μg/ml G418 (Invitrogen) and 100 μg/ml clonNAT (Werner BioAgents).

### SGA screening

The method described in [27] was used. Briefly, the query strain ER*ccw12*Δ was pinned onto a fresh YPD plate at a density of 384 cells per plate. Then, the deletion mutant array was pinned on top of the query cells. The resulting diploids were selected on YPD containing G418 (200 μg/ml) and clonNAT (100 μg/ml). Arrays were then pinned onto sporulation medium. After a 9-day incubation at 23°C spores were pinned onto haploid selection medium (SD supplemented with histidine, arginine and canavanine) to select for growth of *MAT***α **spore progeny. For 2 days meiotic progenies carrying the G418 resistance locus were selected on medium containing G418 at 30°C (SD supplemented with histidine, arginine, canavanine, G418). The G418 and Nat resistant cells were selected on medium containing G418 and clonNAT for 2 days at 30°C (SD supplemented with histidine, arginine, canavanine, G418, nourseothricin). Colony size was scored by eye. Each screen was done in duplicate.

To confirm putative interactions spores were diluted in sterile water, spotted onto solid haploid selection medium and incubated for 2 days at 30°C (selection of the *MAT***α **progeny). Double mutants were then selected as described above. According to colony growth double mutants were scored as synthetic sick (ss) or synthetic lethal (sl) after 2 days of incubation at 30°C.

### Microarray analysis of differential gene expression

SEY6210 and *ccw12*Δ (MEY12A) strains were cultured in YPD supplemented with adenine (39 mg/l) to an OD_600 _of 1. Total RNA was extracted according to [71]. For each strain two pools of three independent RNA preparations were obtained, representing two biological replicates each. Concentration of total RNA was measured at 260 nm and sample quality was checked using RNA Nano Labchips in a Bioanalyzer 2100B (Agilent Technologies, Palo Alto, CA). Double stranded cDNA was synthesized from 5 μg of total RNA using "One-cycle cDNA Synthesis Kit" (Affymetrix, Santa Clara, CA). After cDNA purification using the "GeneChip Sample Cleanup Module" (Affymetrix), this DNA was used as template for the *in vitro *transcription using the "IVT Labeling kit" (Affymetrix) to obtain the biotin labelled cRNA. The obtained cRNA was fragmented and hybridized to the Affymetrix GeneChip^® ^Yeast Genome S98 array for 16 h at 45°C. Hybridized microarrays were washed and stained with a streptavidin-phycoerythrin conjugate in a "GeneChip^® ^Fluidics Station 450". All these procedures were carried out as suggested by the manufacturer. Hybridized cRNA was finally identified by the fluorescence signal in a "GeneChip^® ^3000" scanner. The files generated from the scanning were converted to gene expression signals using the GCOS software (Affymetrix, [72]). Data from the two mutant samples were compared against data from the two SEY6210 samples, obtaining a total of four comparisons. Genes with at least two Present values as "Detection call" and four Increase or Decrease values as "Signal call" were selected for further analysis. The Signal Log Ratio was obtained as the mean of the Signal Log Ratio obtained in each of the four comparisons, and genes with Signal Ratio >2 or < 0.5 were considered for further analysis. Clustering and visualization of the data was performed using the MeV software (TIGR) [73].

### Quantitative RT-PCR assays

SEY6210 and *ccw12*Δ (MEY12A) strains were cultured in YPD supplemented with adenine (39 mg/l) to an OD_600 _of 1. Total RNA was isolated from 5 × 10^7 ^cells using the RNeasy MINI kit (mechanical disruption protocol; QIAGEN, Hilden, Germany). On-column DNase digestion of the samples was performed following manufacturer's instructions. From each strain, two biological replicates were processed. Concentration of total RNA was determined by measuring the absorbance at 260 nm. RNA purity and integrity were assessed using the RNA Nano Labchips in an Agilent 2100B Bioanalyzer (Agilent Technologies, Palo Alto, CA). First strand cDNAs were synthesized from 1 μg of total RNA in 20 μl final volume, using the High Capacity RNA-to-cDNA Master Mix (Applied Biosystems) following the recommendations of the manufacturer. To exclude the presence of genomic DNA, reactions were performed in the absence of reverse transcriptase. Real time quantitative PCR assays were carry out in an ABI 7900HT Fast Real-Time PCR instrument (Applied Biosystems, Foster city, CA) using standard PCR conditions. Duplicates of all reactions were analysed. Quantification of *BCK1, CHS3, ECM13, EDE1, FIG1, FUS1, MLP1, PFD1, PRM10, PRM3, SLA1, SLT2*, and *YPS5 *was performed using the "FastStart Universal SYBR Green Master (ROX) (Roche Applied Science)". Based on ORF sequences provided in SGD (http://www.yeastgenome.org/), gene-specific primers were designed using "Primer Express^® ^Software" (Applied Biosystems). Primer sequences will be provided upon request. For quantification, the abundance of each transcript in the mutant was determined relative to the standard 18S ribosomal RNA and with respect to the wt by using the Comparative Ct Method. Following this approach, the two RNA samples from the mutant were compared to each of the two samples from the wt strain, thus obtaining a total of four comparisons (four ratios). Final data for each transcript was expressed as a media of these four ratios with their corresponding standard deviation.

### Test for Calcofluor white (CW) sensitivity

Strains were grown in YPD medium to exponential phase. A total of 10^8 ^cells were collected and suspended in 0.1 ml of sterile water. 10-fold serial dilutions were prepared. 3 μl of each dilution were spotted onto YPD plates containing 25 μg/ml CW. After two days of incubation at 30°C, growth was scored,

### Preparation of cell extracts and Western blot analyses

Yeast strains were grown in YPD medium to 2 × 10^7 ^cells/ml. In the case of stress induction experiments, log-phase cultures were treated with CW (40 μg/ml for 1 hour) or exposed to elevated temperature (37°C for 3 h). 1 × 10^7 ^cells were collected and cell pellets frozen in liquid nitrogen. 20 μl of SDS sample buffer (60 mM Tris-Cl pH 6.8, 2% SDS, 10% glycerol, 5% β-mercaptoethanol, 0.01% bromophenol blue) were added to the frozen cells. Samples were boiled for 5 min to extract proteins.

Protein extracts were analysed by SDS-PAGE (8% PA gels) under reducing conditions and transferred to nitrocellulose. Anti-Phospho-p44/42 MAP Kinase polyclonal antibodies (9101 - Cell Signalling) were used at 1:3,000 dilution; anti-Pfk1p polyclonal antibodies (gift of Prof. Juergen Heinisch) were used at 1:30,000 dilution. Peroxidase-conjugated anti-rabbit secondary antibodies (Jackson Immunoresearch) were used at a 1:10,000 dilution. Protein-antibody complexes were visualized by enhanced chemiluminescence using the GE Healthcare system.

### Pheromone treatment

Exponentially growing yeast cells (5 × 10^6 ^cells/ml YPD) were treated with 20 μg/ml α-factor (GenScript) or mock treated. At indicated times, cells were analysed by phase-contrast microscopy and scored for budding and shmoo formation by counting at least 200 cells after mild sonication.

### Fluorescence microscopy

Yeast strains transformed with plasmid pCCW12-GFP were grown under selective conditions. Cells were collected by centrifugation, washed twice with PBS, and incubated on ice for at least 15 min before further processing. The cells were examined as wet mounts using a BX60 microscope (Olympus, Melville, NY) and a DC290 digital photo camera (Eastman Kodak, Rochester, NY) or an Eclipse 90i microscope (Nikon, Tokyo, Japan) equipped with epifluorescence, Nomarski optics, and a Hamamatsu ORCA-ER device camera (Nuhsbaum, McHenry, IL). Cell viability was analysed by methylene blue staining [74]. For DNA staining, 8.3 μg/ml 4,6-diamidine-phenylindole (DAPI) was used.

To determine cell sizes, differential interference contrast pictures of log-phase cells were taken. The ratio of the length of the long and short cell axis was determined. For average values at least 180 cells per strain were surveyed.

## Authors' contributions

ER carried out the molecular genetic and physiological studies, participated in the microarray data analyses. HP carried out the microarray experiments, JGC performed the microarray statistical analysis and qRT-PCR; and CN participated in the SGA set up. ER and SS drafted the manuscript. LP, MA, JA and SS conceived the study, and participated in its design and coordination, and helped to draft the manuscript. All authors read and approved the final manuscript.

## Supplementary Material

Additional file 1 *ccw12*Δ microarray dataset Click here for file

Additional file 2 Genes up- and down-regulated in *ccw12*Δ mutant Click here for file

Additional file 3 Northern blot analysis of *ECM13 *and *PIR3 *expression in *ccw12*Δ cells Click here for file

## References

[B1] Nguyen TH Fleet GH Rogers PL Composition of the cell walls of several yeast species Appl Microbiol Biotechnol 1998 50 206 212 10.1007/s002530051278 9763691

[B2] Smits GJ Kapteyn JC Van Den EH Klis FM Cell wall dynamics in yeast Curr Opin Microbiol 1999 2 348 352 10.1016/S1369-5274(99)80061-7 10458981

[B3] Klis FM Mol P Hellingwerf K Brul S Dynamics of cell wall structure in *Saccharomyces cerevisiae* FEMS Microbiol Rev 2002 26 239 256 10.1111/j.1574-6976.2002.tb00613.x 12165426

[B4] Kollár R Reinhold BB Petráková E Yeh HJ Ashwell G Drgonová J Kapteyn JC Klis FM Cabib E Architecture of the yeast cell wall. β-1,6-glucan interconnects mannoprotein, β-1,3-glucan, and chitin J Biol Chem 1997 272 17762 17775 9211929 10.1074/jbc.272.28.17762

[B5] Cabib E Durán A Synthase III-dependent chitin is bound to different acceptors depending on location on the cell wall of budding yeast J Biol Chem 2005 280 9170 9 10.1074/jbc.M414005200 15637060

[B6] Cappellaro C Mrsa V Tanner W New potential cell wall glucanases of *Saccharomyces cerevisiae *and their involvement in mating J Bacteriol 1998 180 5030 7 9748433 10.1128/jb.180.19.5030-5037.1998 PMC107536

[B7] Mrsa V Ecker M Strahl-Bolsinger S Nimtz M Lehle L Tanner W Deletion of new covalently linked cell wall glycoproteins alters the electrophoretic mobility of phosphorylated wall components of *Saccharomyces cerevisiae* J Bacteriol 1999 181 3076 3086 10322008 10.1128/jb.181.10.3076-3086.1999 PMC93762

[B8] Ecker M Deutzmann R Lehle L Mrsa V Tanner W Pir proteins of *Saccharomyces cerevisiae *are attached to β-1,3-glucan by a new protein-carbohydrate linkage J Biol Chem 2006 281 11523 11529 10.1074/jbc.M600314200 16495216

[B9] De Groot PW Hellingwerf KJ Klis FM Genome-wide identification of fungal GPI proteins Yeast 2003 20 781 796 10.1002/yea.1007 12845604

[B10] Yin QY de Groot PW de Jong L Klis FM De Koster CG Mass spectrometric quantitation of covalently bound cell wall proteins in *Saccharomyces cerevisiae* FEMS Yeast Res 2007 7 887 96 10.1111/j.1567-1364.2007.00272.x 17617218 PMC2040195

[B11] Ragni E Sipiczki M Strahl S Characterization of Ccw12p, a major key player in cell wall stability of *Saccharomyces cerevisiae* Yeast 2007 24 309 19 10.1002/yea.1465 17315267

[B12] Hagen I Ecker M Lagorce A Francois JM Sestak S Rachel R Grossmann G Hauser NC Hoheisel JD Tanner W Strahl S Sed1p and Srl1p are required to compensate for cell wall instability in *Saccharomyces cerevisiae *mutants defective in multiple GPI-anchored mannoproteins Mol Microbiol 2004 52 1413 1425 10.1111/j.1365-2958.2004.04064.x 15165243

[B13] Shankarnarayan S Malone CL Deschenes RJ Fassler JS Modulation of yeast Sln1 kinase activity by the *CCW12 *cell wall protein J Biol Chem 2008 283 1962 73 10.1074/jbc.M805860200 18048366 PMC2892218

[B14] Cabib E Blanco N Grau C Rodríguez-Peña JM Arroyo J Crh1p and Crh2p are required for the cross-linking of chitin to beta(1-6)glucan in the *Saccharomyces cerevisiae *cell wall Mol Microbiol 2007 63 921 35 10.1111/j.1365-2958.2006.05565.x 17302808

[B15] Lagorce A Hauser NC Labourdette D Rodriguez C Martin-Yken H Arroyo J Hoheisel JD Francois J Genome-wide analysis of the response to cell wall mutations in the yeast *Saccharomyces cerevisiae* J Biol Chem 2003 278 20345 20357 10.1074/jbc.M211604200 12644457

[B16] García R Bermejo C Grau C Pérez R Rodríguez-Peña JM Francois J Nombela C Arroyo J The global transcriptional response to transient cell wall damage in *Saccharomyces cerevisiae *and its regulation by the cell integrity signalling pathway J Biol Chem 2004 279 15183 95 14739279 10.1074/jbc.M312954200

[B17] Boorsma A de Nobel H ter Riet B Bargmann B Brul S Hellingwerf KJ Klis FM Characterization of the transcriptional response to cell wall stress in *Saccharomyces cerevisiae* Yeast 2004 21 413 27 10.1002/yea.1109 15116342

[B18] Jung US Levin DE Genome-wide analysis of gene expression regulated by the yeast cell wall integrity signalling pathway Mol Microbiol 1999 34 1049 1057 10.1046/j.1365-2958.1999.01667.x 10594829

[B19] De Groot PW Ruiz C Vazquez de Aldana C Dueñas E Cid V Del Rey F Rodríguez Peña JM Pérez P Andel A Caubín J Arroyo J García JC Gil C Molina M García LJ Nombela C Klis FM A genomic approach for the identification and classification of genes involved in cell wall formation and its regulation in *Saccharomyces cerevisiae* Comp Funct Genom 2001 2 124 142 10.1002/cfg.85 PMC2447203 18628907

[B20] Zu T Verna J Ballester R Mutations in *WSC *genes for putative stress receptors result in sensitivity to multiple stress conditions and impairment of Rlm1-dependent gene expression in *Saccharomyces cerevisiae* Mol Genet Genomics 2001 266 142 155 10.1007/s004380100537 11589572

[B21] Rajavel M Philip B Buehrer BM Errede B Levin DE Mid2 is a putative sensor for cell integrity signaling in *Saccharomyces cerevisiae* Mol Cell Biol 1999 19 3969 3976 10330137 10.1128/mcb.19.6.3969 PMC104356

[B22] Parrish WR Stefan CJ Emr SD PtdIns(3)P accumulation in triple lipid-phosphatase-deletion mutants triggers lethal hyperactivation of the Rho1p/Pkc1p cell-integrity MAP kinase pathway J Cell Sci 2005 118 5589 601 10.1242/jcs.02649 16306222

[B23] Levin DE Cell wall integrity signalling in *Saccharomyces cerevisiae* Microbiol Mol Biol Rev 2005 69 262 91 Review 10.1128/MMBR.69.2.262-291.2005 15944456 PMC1197416

[B24] Valdivieso MH Ferrario L Vai M Duran A Popolo L Chitin synthesis in a *gas1 *mutant of *Saccharomyces cerevisiae* J Bacteriol 2000 182 4752 7 10.1128/JB.182.17.4752-4757.2000 10940014 PMC111350

[B25] Bulawa CE Genetics and molecular biology of chitin synthesis in fungi Annu Rev Microbiol 1993 47 505 34 Review 10.1146/annurev.mi.47.100193.002445 8257107

[B26] Carotti C Ferrario L Roncero C Valdivieso MH Duran A Popolo L Maintenance of cell integrity in the *gas1 *mutant of *Saccharomyces cerevisiae *requires the Chs3p-targeting and activation pathway and involves an unusual Chs3p localization Yeast 2002 19 1113 24 10.1002/yea.905 12237852

[B27] Tong AH Evangelista M Parsons AB Xu H Bader GD Pagé N Robinson M Raghibizadeh S Hogue CW Bussey H Andrews B Tyers M Boone C Systematic genetic analysis with ordered arrays of yeast deletion mutants Science 2001 294 2364 8 10.1126/science.1065810 11743205

[B28] Tong AH Lesage G Bader GD Ding H Xu H Xin X Young J Berriz GF Brost RL Chang M Chen Y Cheng X Chua G Friesen H Goldberg DS Haynes J Humphries C He G Hussein S Ke L Krogan N Li Z Levinson JN Lu H Ménard P Munyana C Parsons AB Ryan O Tonikian R Roberts T Sdicu AM Shapiro J Sheikh B Suter B Wong SL Zhang LV Zhu H Burd CG Munro S Sander C Rine J Greenblatt J Peter M Bretscher A Bell G Roth FP Brown GW Andrews B Bussey H Boone C Global mapping of the yeast genetic network Science 2004 303 808 813 10.1126/science.1091317 14764870

[B29] Green R Lesage G Sdicu AM Ménard P Bussey H A synthetic analysis of the *Saccharomyces cerevisiae *stress sensor Mid2p, and identification of a Mid2p-interacting protein, Zeo1p, that modulates the PKC1-MPK1 cell integrity pathway Microbiology 2003 149 2487 99 10.1099/mic.0.26471-0 12949174

[B30] Lesage G Sdicu AM Ménard P Shapiro J Hussein S Bussey H Analysis of beta-1,3-glucan assembly in *Saccharomyces cerevisiae *using a synthetic interaction network and altered sensitivity to caspofungin Genetics 2004 167 35 49 10.1534/genetics.167.1.35 15166135 PMC1470839

[B31] Costanzo M Baryshnikova A Bellay J Kim Y Spear ED Sevier CS Ding H Koh JL Toufighi K Mostafavi S Prinz J St Onge RP VanderSluis B Makhnevych T Vizeacoumar FJ Alizadeh S Bahr S Brost RL Chen Y Cokol M Deshpande R Li Z Lin ZY Liang W Marback M Paw J San Luis BJ Shuteriqi E Tong AH van Dyk N Wallace IM Whitney JA Weirauch MT Zhong G Zhu H Houry WA Brudno M Ragibizadeh S Papp B Pál C Roth FP Giaever G Nislow C Troyanskaya OG Bussey H Bader GD Gingras AC Morris QD Kim PM Kaiser CA Myers CL Andrews BJ Boone C The genetic landscape of a cell Science 2010 327 425 31 10.1126/science.1180823 20093466 PMC5600254

[B32] Trilla JA Cos T Duran A Roncero C Characterization of *CHS4 *(*CAL2*), a gene of *Saccharomyces cerevisiae *involved in chitin biosynthesis and allelic to *SKT5 *and *CSD4* Yeast 1997 13 795 807 10.1002/(SICI)1097-0061(199707)13:9<795::AID-YEA139>3.0.CO;2-L 9234668

[B33] DeMarini DJ Adams AE Fares H De Virgilio C Valle G Chuang JS Pringle JR A septin-based hierarchy of proteins required for localized deposition of chitin in the *Saccharomyces cerevisiae *cell wall J Cell Biol 1997 139 75 93 10.1083/jcb.139.1.75 9314530 PMC2139831

[B34] Santos B Snyder M Targeting of chitin synthase 3 to polarized growth sites in yeast requires Chs5p and Myo2p J Cell Biol 1997 136 95 110 10.1083/jcb.136.1.95 9008706 PMC2132460

[B35] Trilla JA Durán A Roncero C Chs7p, a new protein involved in the control of protein export from the endoplasmic reticulum that is specifically engaged in the regulation of chitin synthesis in *Saccharomyces cerevisiae* J Cell Biol 1999 145 1153 63 10.1083/jcb.145.6.1153 10366589 PMC2133151

[B36] Lagorce A Le Berre-Anton V Aguilar-Uscanga B Martin-Yken H Dagkessamanskaia A François J Involvement of *GFA1*, which encodes glutamine-fructose-6-phosphate amidotransferase, in the activation of the chitin synthesis pathway in response to cell-wall defects in *Saccharomyces cerevisiae* Eur J Biochem 2002 269 1697 707 10.1046/j.1432-1327.2002.02814.x 11895440

[B37] Lam KK Davey M Sun B Roth AF Davis NG Conibear E Palmitoylation by the DHHC protein Pfa4 regulates the ER exit of Chs3 J Cell Biol 2006 174 19 25 10.1083/jcb.200602049 16818716 PMC2064155

[B38] Schmidt M Strenk ME Boyer MP Fritsch BJ Importance of cell wall mannoproteins for septum formation in *Saccharomyces cerevisiae* Yeast 2005 22 715 23 10.1002/yea.1242 16034811

[B39] Kojima H Hashimoto H Yoda K Interaction among the subunits of Golgi membrane mannosyltransferase complexes of the yeast *Saccharomyces cerevisiae* Biosci Biotechnol Biochem 1999 63 1970 6 10.1271/bbb.63.1970 10635561

[B40] Bermejo C Rodríguez E García R Rodríguez-Peña JM Rodríguez de la Concepción ML Rivas C Arias P Nombela C Posas F Arroyo J The sequential activation of the yeast *HOG *and *SLT2 *pathways is required for cell survival to cell wall stress Mol Biol Cell 2008 19 1113 24 10.1091/mbc.E07-08-0742 18184748 PMC2262984

[B41] García R Rodríguez-Peña JM Bermejo C Nombela C Arroyo J The high osmotic response and cell wall integrity pathways cooperate to regulate transcriptional responses to zymolyase-induced cell wall stress in *Saccharomyces cerevisiae* J Biol Chem 2009 284 10901 11 19234305 10.1074/jbc.M808693200 PMC2667776

[B42] Piao HL Machado IM Payne GS NPFXD-mediated endocytosis is required for polarity and function of a yeast cell wall stress sensor Mol Biol Cell 2007 18 57 65 10.1091/mbc.E06-08-0721 17065552 PMC1751320

[B43] Huttenhower C Myers CL Hibbs MA Troyanskaya OG Computational analysis of the yeast proteome: understanding and exploiting functional specificity in genomic data Methods Mol Biol 2009 548 273 93 full_text 19521830 10.1007/978-1-59745-540-4_15

[B44] Smits GJ van den Ende H Klis FM Differential regulation of cell wall biogenesis during growth and development in yeast Microbiology 2001 147 781 794 11283274 10.1099/00221287-147-4-781

[B45] Heinisch JJ Lorberg A Schmitz HP Jacoby JJ The protein kinase C-mediated MAP kinase pathway involved in the maintenance of cellular integrity in *Saccharomyces cerevisiae* Mol Microbiol 1999 32 671 80 10.1046/j.1365-2958.1999.01375.x 10361272

[B46] Rep M Krantz M Thevelein JM Hohmann S The transcriptional response of *Saccharomyces cerevisiae *to osmotic shock. Hot1p and Msn2p/Msn4p are required for the induction of subsets of high osmolarity glycerol pathway-dependent genes J Biol Chem 2000 275 8290 300 10.1074/jbc.275.12.8290 10722658

[B47] Roberts CJ Nelson B Marton MJ Stoughton R Meyer MR Bennett HA He YD Dai H Walker WL Hughes TR Tyers M Boone C Friend SH Signaling and circuitry of multiple MAPK pathways revealed by a matrix of global gene expression profiles Science 2000 287 873 80 10.1126/science.287.5454.873 10657304

[B48] Brown JL Bussey H The yeast *KRE9 *gene encodes an O glycoprotein involved in cell surface beta-glucan assembly Mol Cell Biol 1993 13 6346 56 8413233 10.1128/mcb.13.10.6346-6356.1993 PMC364693

[B49] Gagnon-Arsenault I Tremblay J Bourbonnais Y Fungal yapsins and cell wall: a unique family of aspartic peptidases for a distinctive cellular function FEMS Yeast Res 2006 6 966 78 10.1111/j.1567-1364.2006.00129.x 17042746

[B50] Burke J Lipari F Igdoura S Herscovics A The *Saccharomyces cerevisiae *processing alpha 1,2-mannosidase is localized in the endoplasmic reticulum, independently of known retrieval motifs Eur J Cell Biol 1996 70 298 305 8864657

[B51] Herscovics A Orlean P Glycoprotein biosynthesis in yeast FASEB J 1993 7 540 50 8472892 10.1096/fasebj.7.6.8472892

[B52] Protchenko O Rodriguez-Suarez R Androphy R Bussey H Philpott CC A screen for genes of heme uptake identifies the FLC family required for import of FAD into the endoplasmic reticulum J Biol Chem 2006 281 21445 57 10.1074/jbc.M512812200 16717099

[B53] Hughes TR Marton MJ Jones AR Roberts CJ Stoughton R Armour CD Bennett HA Coffey E Dai H He YD Kidd MJ King AM Meyer MR Slade D Lum PY Stepaniants SB Shoemaker DD Gachotte D Chakraburtty K Simon J Bard M Friend SH Functional discovery via a compendium of expression profiles Cell 2000 102 109 26 10.1016/S0092-8674(00)00015-5 10929718

[B54] Watanabe M Watanabe D Nogami S Morishita S Ohya Y Comprehensive and quantitative analysis of yeast deletion mutants defective in apical and isotropic bud growth Curr Genet 2009 55 365 80 10.1007/s00294-009-0251-0 19466415

[B55] Delley PA Hall MN Cell wall stress depolarizes cell growth via hyperactivation of *RHO1* J Cell Biol 1999 147 163 74 10.1083/jcb.147.1.163 10508863 PMC2164985

[B56] Arkowitz RA Responding to attraction: chemotaxis and chemotropism in Dictyostelium and yeast Trends Cell Biol 1999 9 20 7 10.1016/S0962-8924(98)01412-3 10087613

[B57] Chant J Cell polarity in yeast Annu Rev Cell Dev Biol 1999 15 365 91 10.1146/annurev.cellbio.15.1.365 10611966

[B58] Trueheart J Fink GR The yeast cell fusion protein FUS1 is O-glycosylated and spans the plasma membrane Proc Natl Acad Sci USA 1989 86 9916 20 10.1073/pnas.86.24.9916 2690078 PMC298613

[B59] Hutzler F Gerstl R Lommel M Strahl S Protein N-glycosylation determines functionality of the *Saccharomyces cerevisiae *cell wall integrity sensor Mid2p Mol Microbiol 2008 68 1438 49 10.1111/j.1365-2958.2008.06243.x 18410496

[B60] Lesage G Shapiro J Specht CA Sdicu AM Ménard P Hussein S Tong AH Boone C Bussey H An interactional network of genes involved in chitin synthesis in *Saccharomyces cerevisiae* BMC Genet 2005 16 8 10.1186/1471-2156-6-8 PMC554099 15715908

[B61] Sheikh-Hamad D Gustin MC MAP kinases and the adaptive response to hypertonicity: functional preservation from yeast to mammals Am J Physiol Renal Physiol 2004 287 1102 10 10.1152/ajprenal.00225.2004 15522988

[B62] Zhan XL Deschenes RJ Guan KL Differential regulation of *FUS3 *MAP kinase by tyrosine-specific phosphatases *PTP2/PTP3 *and dual-specificity phosphatase *MSG5 *in *Saccharomyces cerevisiae* Genes Dev 1997 11 1690 702 10.1101/gad.11.13.1690 9224718

[B63] Leza MA Elion EA *POG1*, a novel yeast gene, promotes recovery from pheromone arrest via the G1 cyclin *CLN2* Genetics 1999 151 531 43 9927449 10.1093/genetics/151.2.531 PMC1460478

[B64] Madden K Sheu YJ Baetz K Andrews B Snyder M SBF cell cycle regulator as a target of the yeast PKC-MAP kinase pathway Science 1997 275 1781 4 10.1126/science.275.5307.1781 9065400

[B65] Hadwiger JA Wittenberg C Richardson HE de Barros Lopes M Reed SI A family of cyclin homologs that control the G1 phase in yeast Proc Natl Acad Sci USA 1989 86 6255 9 10.1073/pnas.86.16.6255 2569741 PMC297816

[B66] Oehlen LJ Cross FR G1 cyclins *CLN1 *and *CLN2 *repress the mating factor response pathway at Start in the yeast cell cycle Genes Dev 1994 8 1058 70 10.1101/gad.8.9.1058 7926787

[B67] Sambrook J Fitsch EF Maniatis T Molecular Cloning: A Laboratory Manual 1989 Cold Spring Harbor: Cold Spring Harbor Press

[B68] Robinson JS Klionsky DJ Banta LM Emr SD Protein sorting in *Saccharomyces cerevisiae*: isolation of mutants defective in the delivery and processing of multiple vacuolar hydrolases Mol Cell Biol 1988 8 4936 4948 3062374 10.1128/mcb.8.11.4936-4948.1988 PMC365587

[B69] Brachmann CB Davies A Cost GJ Caputo E Li J Hieter P Boeke JD Designer deletion strains derived from S*accharomyces cerevisiae *S288C: a useful set of strains and plasmids for PCR-mediated gene disruption and other applications Yeast 1998 14 115 32 10.1002/(SICI)1097-0061(19980130)14:2<115::AID-YEA204>3.0.CO;2-2 9483801

[B70] Rothstein RJ One-step gene disruption in yeast Methods Enzymol 1983 101 202 11 full_text 6310324 10.1016/0076-6879(83)01015-0

[B71] Schmitt R Sandermann H Jr Biochemical response of Norway spruce (Picea abies (L.) karst.) towards 14-month exposure to ozone and acid mist: part II--Effects on protein biosynthesis Environ Pollut 1990 64 367 73 10.1016/0269-7491(90)90058-K 15092292

[B72] Wood V Gwilliam R Rajandream MA Lyne M Lyne R Stewart A Sgouros J Peat N Hayles J Baker S Basham D Bowman S Brooks K Brown D Brown S Chillingworth T Churcher C Collins M Connor R Cronin A Davis P Feltwell T Fraser A Gentles S Goble A Hamlin N Harris D Hidalgo J Hodgson G Holroyd S Hornsby T Howarth S Huckle EJ Hunt S Jagels K James K Jones L Jones M Leather S McDonald S McLean J Mooney P Moule S Mungall K Murphy L Niblett D Odell C Oliver K O'Neil S Pearson D Quail MA Rabbinowitsch E Rutherford K Rutter S Saunders D Seeger K Sharp S Skelton J Simmonds M Squares R Squares S Stevens K Taylor K Taylor RG Tivey A Walsh S Warren T Whitehead S Woodward J Volckaert G Aert R Robben J Grymonprez B Weltjens I Vanstreels E Rieger M Schäfer M Müller-Auer S Gabel C Fuchs M Düsterhöft A Fritzc C Holzer E Moestl D Hilbert H Borzym K Langer I Beck A Lehrach H Reinhardt R Pohl TM Eger P Zimmermann W Wedler H Wambutt R Purnelle B Goffeau A Cadieu E Dréano S Gloux S Lelaure V Mottier S Galibert F Aves SJ Xiang Z Hunt C Moore K Hurst SM Lucas M Rochet M Gaillardin C Tallada VA Garzon A Thode G Daga RR Cruzado L Jimenez J Sánchez M del Rey F Benito J Domínguez A Revuelta JL Moreno S Armstrong J Forsburg SL Cerutti L Lowe T McCombie WR Paulsen I Potashkin J Shpakovski GV Ussery D Barrell BG Nurse P The genome sequence of *Schizosaccharomyces pombe* Nature 2002 415 871 80 10.1038/nature724 11859360

[B73] Saeed AI Sharov V White J Li J Liang W Bhagabati N Braisted J Klapa M Currier T Thiagarajan M Sturn A Snuffin M Rezantsev A Popov D Ryltsov A Kostukovich E Borisovsky I Liu Z Vinsavich A Trush V Quackenbush J TM4: a free, open-source system for microarray data management and analysis Biotechniques 2003 34 374 8 12613259 10.2144/03342mt01

[B74] Iida H Yagawa Y Anraku Y Essential role for induced Ca^2+ ^influx followed by [Ca^2+^]i rise in maintaining viability of yeast cells late in the mating pheromone response pathway. A study of [Ca^2+^]i in single *Saccharomyces cerevisiae *cells with imaging of fura-2 J Biol Chem 1990 265 13391 13399 2198292

