# Supplementary material for: The genetic interaction network of CCW12, a Saccharomyces cerevisiae gene required for cell wall integrity during budding and formation of mating projections
Source: BMC Genomics. 2011 Feb 14;12:107. doi: 10.1186/1471-2164-12-107 (PMC3049148; doi:10.1186/1471-2164-12-107)
Supplement: Additional file 2 — Genes up- and down-regulated in ccw12Δ mutant [file 1471-2164-12-107-S2.DOC]

| **Gene symbol** | **ORF** | **Fold change** | **Gene description** |  | |
| --- | --- | --- | --- | --- | --- |
| *ECM13* | YBL043W | **11.71** | Non-essential protein of unknown function. |  | |
| *XBP1* | YIL101C | **8.28** | Transcriptional repressor; binds to promoter of the cyclin genes. |  | |
| *THI11* | YJR156C | **7.86** | Protein involved in synthesis of the hydroxymethylpyrimidine. |  | |
| *ARO10* | YDR380W | **6.96** | Phenylpyruvate decarboxylase. |  | |
| *MMS4* | YBR098W | **6.84** | Subunit of the structure-specific Mms4p-Mus81p endonuclease. |  | |
| *BNA2* | YJR078W | **6.73** | Tryptophan 2,3-dioxygenase. |  | |
| *YBR056w-a* | YBR056W-A | **6.17** | identified by SAGE. |  | |
| *YLR108C* | YLR108C | **5.46** | Hypothetical protein. |  | |
| *SPT10* | YJL127C | **4.92** | Putative histone acetylase. |  | |
| *BNA3* | YJL060W | **4.68** | Arylformamidase. |  | |
| *PDH1* | YPR002W | **4.52** | Mitochondrial protein that participates in respiration. |  | |
| *YBR108W* | YBR108W | **4.52** | Protein interacting with Rsv167p. |  | |
| *SWE1* | YJL187C | **4.44** | Protein kinase that regulates the G2/M transition. |  | |
| *YKL161C* | YKL161C | **4.44** | Mpk1-like protein kinase. |  | |
| *YPS6* | YIR039C | **4.44** | Putative GPI-anchored aspartic protease. |  | |
| *YPS5* | YGL259W | **4.44** | Putative GPI-anchored aspartic protease. |  | |
| *ARO9* | YHR137W | **4.36** | Aromatic aminotransferase. |  | |
| *BBC1* | YJL020C | **4.36** | Protein possibly involved in assembly of actin patches. |  | |
| *SDP1* | YIL113W | **4.36** | Stress-inducible dual-specificity MAP kinase phosphatase. |  | |
| *YBR071W* | YBR071W | **4.36** | Hypothetical protein. |  | |
| *YRO2* | YBR054W | **4.29** | Putative plasma membrane protein of unknown function. |  | |
| *OM45* | YIL136W | **4.21** | Protein of unknown function. |  | |
| *YJR079W* | YJR079W | **4.21** | questionable ORF. |  | |
| *YBR230C* | YBR230C | **4.14** | Hypothetical protein. |  | |
| *THI2* | YBR240C | **4** | Zinc finger protein of the Zn(II)2Cys6 type. |  | |
| *YIL141W* | YIL141W | **4** | questionable ORF. |  | |
| *YTP1* | YNL237W | **4** | Probable type-III integral membrane protein of unknown function. |  | |
| *YJL057C* | YJL057C | **3.93** | probable serine/threonine kinase. |  | |
| *YMR1* | YJR110W | **3.93** | Phosphatidylinositol 3-phosphate [PI(3)P] phosphatase. |  | |
| *HPR5* | YJL092W | **3.86** | DNA helicase and DNA-dependent ATPase . |  | |
| *KIP1* | YBL063W | **3.86** | Kinesin-related motor protein. |  | |
| *MSL1* | YIR009W | **3.86** | U2B component of U2 snRNP. |  | |
| *MGM101* | YJR144W | **3.8** | Protein involved in mitochondrial genome maintenance. |  | |
| *MPM1* | YJL066C | **3.8** | Mitochondrial membrane protein of unknown function. |  | |
| *TOK1* | YJL093C | **3.8** | Outward-rectifier potassium channel of the plasma membrane. |  | |
| *APS3* | YJL024C | **3.73** | Small subunit of the clathrin-associated adaptor complex AP-3. |  | |
| *FBP26* | YJL155C | **3.73** | Fructose-2,6-bisphosphatase. |  | |
| *YIR042C* | YIR042C | **3.73** | Hypothetical protein. |  | |
| *AKL1* | YBR059C | **3.67** | Ser-Thr protein kinase. |  | |
| *LSM1* | YJL124C | **3.67** | Component of small nuclear ribonucleoprotein complexes. |  | |
| *PBS2* | YJL128C | **3.67** | MAP kinase. |  |  |
| *PRM10* | YJL108C | **3.67** | Pheromone-regulated protein. |  | |
| *TIS11* | YLR136C | **3.67** | mRNA-binding protein expressed during iron starvation. |  | |
| *YBR269C* | YBR269C | **3.67** | The authentic protein was localized to the mitochondria. |  | |
| *YBR085c-a* | YBR085C-A | **3.61** | Hypothetical protein. |  |  |
| *YLR149C* | YLR149C | **3.54** | Hypothetical protein. |  |  |
| *FIS1* | YIL065C | **3.48** | Mitochondrial outer membrane prot. involved in membrane fission. |  | |
| *MDV1* | YJL112W | **3.48** | Peripheral protein, cytosolic face of the mito outer membrane. |  | |
| *NTH2* | YBR001C | **3.48** | Neutral trehalase. |  |  |
| *YJR142W* | YJR142W | **3.48** | Hypothetical protein |  |  |
| *ZAP1* | YJL056C | **3.48** | Zinc-regulated transcription factor. |  | |
| *DPB11* | YJL090C | **3.42** | Essential BRCT repeat protein. |  | |
| *ECM4* | YKR076W | **3.42** | Unknown function. |  |  |
| *HIT1* | YJR055W | **3.42** | Protein of unknown function. |  | |
| *SRL3* | YKR091W | **3.42** | Cytoplasmic protein;when overexpressed, suppresses the lethality of a rad53 null mutation. |  | |
| *YLR267W* | YLR267W | **3.42** | Protein of unknown function. |  | |
| *AGP2* | YBR132C | **3.36** | High affinity polyamine permease. |  | |
| *COX16* | YJL003W | **3.36** | Mitochondrial inner membrane protein. |  | |
| *NCA3* | YJL116C | **3.36** | Protein that regulate mitochondrial expression of subunits of the Fo-F1 ATP synthase. |  | |
| *YJR012C* | YJR012C | **3.36** | hypothetical protein. |  |  |
| *FIT2* | YOR382W | **3.31** | Mannoprotein incorporated into the cell wall via a GPI anchor. |  | |
| *HYS2* | YJR006W | **3.31** | DNA polymerase III (delta) 55 kDa subunit. |  | |
| *LSM2* | YBL026W | **3.31** | Component of small nuclear ribonucleoprotein complexes. |  | |
| *PCL1* | YNL289W | **3.31** | Pho85 cyclin of the Pcl1,2-like subfamily. |  | |
| *PIR3* | YKL163W | **3.31** | O-glycosylated covalently-bound cell wall protein. |  | |
| *PRX1* | YBL064C | **3.31** | Mitochondrial peroxiredoxin (1-Cys Prx). |  | |
| *SSY5* | YJL156C | **3.31** | Component of the SPS plasma membrane aminoacid sensor system. |  | |
| *HIR3* | YJR140C | **3.25** | Transcriptional corepressor. |  | |
| *TPM2* | YIL138C | **3.25** | Minor isoform of tropomyosin. |  | |
| *TRL1* | YJL087C | **3.25** | tRNA ligase. |  |  |
| *ECM17* | YJR137C | **3.19** | Sulfite reductase beta subunit. |  | |
| *FYV10* | YIL097W | **3.19** | Protein of unknown function. |  | |
| *GON7* | YJL184W | **3.19** | Protein of unknown function. |  | |
| *PCS60* | YBR222C | **3.19** | Peroxisomal AMP-binding protein. |  | |
| *SGA1* | YIL099W | **3.19** | Intracellular sporulation-specific glucoamylase. |  | |
| *YBR047W* | YBR047W | **3.19** | The authentic, non-tagged protein was localized to the mitochondria. |  | |
| *YLR194C* | YLR194C | **3.19** | Hypothetical protein. |  |  |
| *PIM1* | YBL022C | **3.14** | Mitochondrial ATP-dependent protease . |  | |
| *POG1* | YIL122W | **3.14** | Putative transcriptional activator; promotes recovery from pheromone induced arrest. |  | |
| *PRE3* | YJL001W | **3.14** | 20S proteasome beta-type subunit. |  | |
| *RRN10* | YBL025W | **3.14** | subunit of UAF (upstream activation factor). |  | |
| *VID28* | YIL017C | **3.14** | Protein involved in proteasome-dependent catabolite degradation of fructose-1,6-bisphosphatase |  | |
| *YAK1* | YJL141C | **3.14** | Serine-threonine protein kinase. |  | |
| *ADR1* | YDR216W | **3.08** | Carbon source-responsive zinc-finger transcription factor. |  | |
| *MEF2* | YJL102W | **3.08** | Mitochondrial elongation factor. |  | |
| *NIT2* | YJL126W | **3.08** | Nit protein. |  |  |
| *PRE7* | YBL041W | **3.08** | 20S proteasome beta-type subunit |  | |
| *NHP6B* | YBR089C-A | **3.03** | High-mobility group non-histone chromatin protein. |  | |
| *PEP8* | YJL053W | **3.03** | Vacuolar protein sorting protein. |  | |
| *SLM4* | YBR077C | **3.03** | Component of the EGO complex. |  | |
| *UGA2* | YBR006W | **3.03** | Succinate semialdehyde dehydrogenase. |  | |
| *YJR039W* | YJR039W | **3.03** | Hypothetical protein. |  |  |
| *IML3* | YBR107C | **2.98** | Protein with a role in kinetochore function. |  | |
| *SSA3* | YBL075C | **2.98** | ATPase involved in protein folding and the response to stress. |  | |
| *TPK1* | YJL164C | **2.98** | Subunit of cytoplasmic cAMP-dependent protein kinase. |  | |
| *YBR056W* | YBR056W | **2.98** | Hypothetical protein. |  |  |
| *YJL070C* | YJL070C | **2.98** | Hypothetical protein. |  |  |
| *EMP46* | YLR080W | **2.93** | Integral membrane component of endoplasmic reticulum-derived COPII-coated vesicles. |  | |
| *ISU1* | YPL135W | **2.93** | Conserved protein of the mitochondrial matrix. |  | |
| *VPS55* | YJR044C | **2.93** | Late endosomal protein . |  | |
| *YDR034W-B* | YDR034W-B | **2.93** | Protein of unknown function. |  | |
| *YJL163C* | YJL163C | **2.93** | Hypothetical protein. |  |  |
| *YPS3* | YLR121C | **2.93** | Aspartic protease. |  |  |
| *CHS6* | YJL099W | **2.88** | Protein of unknown function. |  | |
| *EST3* | YIL009C-A | **2.88** | Component of the telomerase holoenzyme. |  | |
| *FSP2* | YJL221C | **2.88** | Protein of unknown function. |  | |
| *MPS3* | YJL019W | **2.88** | Essential integral membrane protein required for spindle pole body duplication and for nuclear fusion. |  | |
| *NCE103* | YNL036W | **2.88** | Carbonic anhydrase. |  |  |
| *SOP4* | YJL192C | **2.88** | suppressor of pma1-7. |  |  |
| *VPS25* | YJR102C | **2.88** | Component of the ESCRT-II complex. |  | |
| *ZTA1* | YBR046C | **2.88** | Zeta-crystallin homolog. |  |  |
| *CDC6* | YJL194W | **2.83** | Essential ATP-binding protein required for DNA replication. |  | |
| *DAL4* | YIR028W | **2.83** | Allantoin permease. |  |  |
| *JSN1* | YJR091C | **2.83** | Member of the Puf family of RNA-binding proteins. |  | |
| *NPL4* | YBR170C | **2.83** | Endoplasmic reticulum and nuclear membrane protein. |  | |
| *NUP85* | YJR042W | **2.83** | Subunit of the Nup84p subcomplex of the nuclear pore complex. |  | |
| *TIM54* | YJL054W | **2.83** | Translocase Inner Membrane. |  | |
| *VPS70* | YJR126C | **2.83** | Protein of unknown function involved in vacuolar protein sorting |  | |
| *YJL055W* | YJL055W | **2.83** | Hypothetical protein. |  |  |
| *CDC27* | YBL084C | **2.78** | Subunit of the Anaphase-Promoting Complex/Cyclosome (APC/C). |  | |
| *GTT1* | YIR038C | **2.78** | ER associated glutathione S-transferase. |  | |
| *PRM5* | YIL117C | **2.78** | Pheromone-regulated protein. |  | |
| *SHP1* | YBL058W | **2.78** | UBX (ubiquitin regulatory X) domain-containing protein. |  | |
| *SUI2* | YJR007W | **2.78** | Alpha subunit of the translation initiation factor eIF2. |  | |
| *YBR197C* | YBR197C | **2.78** | Hypothetical protein. |  |  |
| *YIL024C* | YIL024C | **2.78** | Hypothetical protein. |  |  |
| *DER1* | YBR201W | **2.73** | Endoplasmic reticulum membrane protein, required for ER-associated protein degradation. |  | |
| *ESS1* | YJR017C | **2.73** | Peptidylprolyl-cis/trans-isomerase (PPIase). |  | |
| *ETR1* | YBR026C | **2.73** | 2-enoyl thioester reductase. |  | |
| *YBR184W* | YBR184W | **2.73** | Hypothetical protein. |  |  |
| *YIL042C* | YIL042C | **2.73** | Hypothetical protein. |  |  |
| *YJL185C* | YJL185C | **2.73** | Hypothetical protein. |  |  |
| *DOT5* | YIL010W | **2.69** | Nuclear thiol peroxidase. |  | |
| *GCD14* | YJL125C | **2.69** | Subunit of tRNA (1-methyladenosine) methyltransferase. |  | |
| *KTR4* | YBR199W | **2.69** | Putative mannosyltransferase involved in protein glycosylation. |  | |
| *OCH1* | YGL038C | **2.69** | Mannosyltransferase of the cis-Golgi apparatus. |  | |
| *RTG3* | YBL103C | **2.69** | Basic helix-loop-helix-leucine zipper transcription factor. |  | |
| *SLA1* | YBL007C | **2.69** | Cytoskeletal protein binding protein. |  | |
| *CAR2* | YLR438W | **2.64** | L-ornithine transaminase (OTAse). |  | |
| *MED8* | YBR193C | **2.64** | Member of RNA Polymerase II transcriptional regulation mediator. |  | |
| *NUP170* | YBL079W | **2.64** | Abundant subunit of the nuclear pore complex (NPC). |  | |
| *REC107* | YJR021C | **2.64** | Protein involved in early stages of meiotic recombination. |  | |
| *TRK1* | YJL129C | **2.64** | Component of the Trk1p-Trk2p potassium transport system. |  | |
| *YHC3* | YJL059W | **2.64** | Vacuolar membrane protein. |  | |
| *APL1* | YJR005W | **2.59** | Beta-adaptin. |  |  |
| *EDE1* | YBL047C | **2.59** | Key endocytic protein . |  | |
| *GUT2* | YIL155C | **2.59** | Mitochondrial glycerol-3-phosphate dehydrogenase. |  | |
| *PRY3* | YJL078C | **2.59** | Protein of unknown function. |  | |
| *SHE1* | YBL031W | **2.59** | Cytoskeletal protein of unknown function. |  | |
| *TES1* | YJR019C | **2.59** | Peroxisomal acyl-CoA thioesterase. |  | |
| *YBL107C* | YBL107C | **2.59** | Hypothetical protein. |  |  |
| *YBR241C* | YBR241C | **2.59** | Hypothetical protein. |  |  |
| *YHR138C* | YHR138C | **2.59** | Homologous to PBI2. |  |  |
| *YJR080C* | YJR080C | **2.59** | The authentic, non-tagged protein was localized to the mitochondria. |  | |
| *ARP4* | YJL081C | **2.55** | Nuclear actin-related protein involved in chromatin remodeling. |  | |
| *ICS2* | YBR157C | **2.55** | Protein of unknown function. |  | |
| *IDH1* | YNL037C | **2.55** | Subunit of mitochondrial NAD(+)-dependent isocitrate dehydrogenase. |  | |
| *KRE9* | YJL174W | **2.55** | Glycoprotein involved in cell wall beta-glucan assembly. |  | |
| *PRK1* | YIL095W | **2.55** | Protein serine/threonine kinase. |  | |
| *RAV1* | YJR033C | **2.55** | Subunit of the RAVE complex (Rav1p, Rav2p, Skp1p). |  | |
| *RDH54* | YBR073W | **2.55** | DNA-dependent ATPase. |  |  |
| *UMP1* | YBR173C | **2.55** | Short-lived chaperone required for maturation of 20S proteasome. |  | |
| *YBR053C* | YBR053C | **2.55** | Hypothetical protein. |  |  |
| *YBR137W* | YBR137W | **2.55** | Hypothetical protein. |  |  |
| *YBR259W* | YBR259W | **2.55** | Hypothetical protein. |  |  |
| *YLR077W* | YLR077W | **2.55** | The authentic, non-tagged protein was localized to the mitochondria. |  | |
| *APL3* | YBL037W | **2.51** | Alpha-adaptin. |  |  |
| *AXL2* | YIL140W | **2.51** | Integral plasma membrane protein. |  | |
| *BCK1* | YJL095W | **2.51** | Mitogen-activated protein (MAP) kinase. |  | |
| *GEF1* | YJR040W | **2.51** | Chloride channel localized to late- or post-Golgi vesicles. |  | |
| *IRR1* | YIL026C | **2.51** | Subunit of the cohesin complex. |  | |
| *KHA1* | YJL094C | **2.51** | Putative K+/H+ antiporter. |  |  |
| *NAS2* | YIL007C | **2.51** | Protein with similarity to the p27 subunit of mammalian proteasome modulator. |  | |
| *PFD1* | YJL179W | **2.51** | Subunit of heterohexameric prefoldin. |  | |
| *RIB7* | YBR153W | **2.51** | Diaminohydroxyphoshoribosylaminopyrimidine deaminase. |  | |
| *SWC5* | YBR231C | **2.51** | Protein of unknown function. |  | |
| *YBR280C* | YBR280C | **2.51** | Hypothetical protein. |  |  |
| *YJR054W* | YJR054W | **2.51** | Vacuolar protein of unknown function. |  | |
| *YJR107W* | YJR107W | **2.51** | Hypothetical protein. |  |  |
| *ACO1* | YLR304C | **2.46** | Aconitase. |  |  |
| *APS2* | YJR058C | **2.46** | Small subunit of the clathrin-associated adaptor complex AP-2. |  | |
| *ATG8* | YBL078C | **2.46** | Protein required for autophagy. |  | |
| *CIT1* | YNR001C | **2.46** | Citrate synthase. |  |  |
| *ECM31* | YBR176W | **2.46** | Ketopantoate hydroxymethyltransferase. |  | |
| *HOR2* | YER062C | **2.46** | One redundant DL-glycerol-3-phosphatase. |  | |
| *VRP1* | YLR337C | **2.46** | Proline-rich, actin-associated protein. |  | |
| *YJL037W* | YJL037W | **2.46** | Hypothetical protein. |  |  |
| *YJL147C* | YJL147C | **2.46** | Hypothetical protein. |  |  |
| *YMR002W* | YMR002W | **2.46** | Hypothetical protein. |  |  |
| *BET1* | YIL004C | **2.42** | Type II membrane protein. |  | |
| *BUD4* | YJR092W | **2.42** | Protein involved in bud-site selection. |  | |
| *CDC11* | YJR076C | **2.42** | Component of the septin ring of the mother-bud neck. |  | |
| *EXO84* | YBR102C | **2.42** | Protein with dual roles in spliceosome assembly and exocytosis. |  | |
| *GAL7* | YBR018C | **2.42** | Galactose-1-phosphate uridyl transferase. |  | |
| *HSP26* | YBR072W | **2.42** | Small heat shock protein with chaperone activity. |  | |
| *ICT1* | YLR099C | **2.42** | Protein of unknown function. |  | |
| *MET1* | YKR069W | **2.42** | S-adenosyl-L-methionine uroporphyrinogen III transmethylase. |  | |
| *RAD7* | YJR052W | **2.42** | Protein that recognizes and binds damaged DNA. |  | |
| *SIM1* | YIL123W | **2.42** | Protein of the SUN family (Sim1p, Uth1p, Nca3p, Sun4p). |  | |
| *YBR062C* | YBR062C | **2.42** | Hypothetical protein. |  |  |
| *YJL046W* | YJL046W | **2.42** | Hypothetical protein. |  |  |
| *YJL132W* | YJL132W | **2.42** | Hypothetical protein. |  |  |
| *YJR096W* | YJR096W | **2.42** | Protein with similarity to aldo-keto reductases. |  | |
| *YKR049C* | YKR049C | **2.42** | The authentic, non-tagged protein was localized to the mito. |  | |
| *YUR1* | YJL139C | **2.42** | Mannosyltransferase of the KTR1 family. |  | |
| *AYR1* | YIL124W | **2.38** | NADPH-dependent 1-acyl dihydroxyacetone phosphate reductase. |  | |
| *BIR1* | YJR089W | **2.38** | Protein involved in cell cycle regulation/chromosome segregation. |  | |
| *CHS3* | YBR023C | **2.38** | Chitin synthase III. |  |  |
| *DLS1* | YJL065C | **2.38** | Subunit of ISW2/yCHRAC chromatin accessibility complex. |  | |
| *ECM21* | YBL101C | **2.38** | Protein of unknown function. |  | |
| *ELO1* | YJL196C | **2.38** | Elongase I, medium-chain acyl elongase. |  | |
| *FAT1* | YBR041W | **2.38** | Fatty acid transporter; very long-chain fatty acyl-CoA synthetase. |  | |
| *FUN34* | YNR002C | **2.38** | Putative transmembrane protein, involved in ammonia production. |  | |
| *JEM1* | YJL073W | **2.38** | DnaJ-like chaperone. |  | |
| *NIT1* | YIL164C | **2.38** | Nitrilase. |  |  |
| *ROX3* | YBL093C | **2.38** | RNA polymerase II holoenzyme component. |  | |
| *SDS24* | YBR214W | **2.38** | S. cerevisiae homolog of the S. pombe Sds23 protein. |  | |
| *SNL1* | YIL016W | **2.38** | Protein of unknown function. |  | |
| *YBR007C* | YBR007C | **2.38** | Hypothetical protein. |  |  |
| *YBR138C* | YBR138C | **2.38** | Cytoplasmic protein of unknown function. |  | |
| *YIL166C* | YIL166C | **2.38** | Hypothetical protein, member of the Dal5p subfamily. |  | |
| *YJL103C* | YJL103C | **2.38** | Hypothetical protein. |  |  |
| *YNL134C* | YNL134C | **2.38** | similarity to C.carbonum toxD gene. |  | |
| *YOR052C* | YOR052C | **2.38** | Hypothetical protein. |  |  |
| *CCT3* | YJL014W | **2.34** | Subunit of the cytosolic chaperonin Cct ring complex. |  | |
| *FAR1* | YJL157C | **2.34** | Cyclin-dependent kinase inhibitor. |  | |
| *FKH1* | YIL131C | **2.34** | Transcription factor of the forkhead family. |  | |
| *HBT1* | YDL223C | **2.34** | Substrate of the Hub1p ubiquitin-like protein. |  | |
| *HEK2* | YBL032W | **2.34** | RNA binding protein with similarity to hnRNP-K. |  | |
| *NCE101* | YJL205C | **2.34** | Protein of unknown function. |  | |
| *PCA1* | YBR295W | **2.34** | P-type metal-transporting ATPase. |  | |
| *PRY2* | YKR013W | **2.34** | Protein of unknown function. |  | |
| *PSF2* | YJL072C | **2.34** | Subunit of the GINS complex (Sld5p. |  | |
| *PTK2* | YJR059W | **2.34** | Putative serine/threonine protein kinase . |  | |
| *SNA3* | YJL151C | **2.34** | Integral membrane protein localized to vacuolar vesicles. |  | |
| *SVS1* | YPL163C | **2.34** | Cell wall and vacuolar protein. |  | |
| *TAH11* | YJR046W | **2.34** | DNA replication licensing factor. |  | |
| *UBP13* | YBL067C | **2.34** | Putative ubiquitin-specific protease. |  | |
| *YBL036C* | YBL036C | **2.34** | Single-domain racemase. |  |  |
| *YHR209W* | YHR209W | **2.34** | Putative S-adenosylmethionine-dependent methyltransferase. |  | |
| *YIL087C* | YIL087C | **2.34** | Hypothetical protein |  |  |
| *YJL149W* | YJL149W | **2.34** | Hypothetical protein. |  |  |
| *YJL217W* | YJL217W | **2.34** | Hypothetical protein |  |  |
| *ARC15* | YIL062C | **2.3** | Subunit of the ARP2/3 complex. |  | |
| *BEM1* | YBR200W | **2.3** | Protein containing SH3-domains. |  | |
| *CCT8* | YJL008C | **2.3** | Subunit of the cytosolic chaperonin Cct ring complex. |  | |
| *CHS2* | YBR038W | **2.3** | Chitin synthase II. |  |  |
| *DAL5* | YJR152W | **2.3** | Allantoin permease. |  |  |
| *DEM1* | YBR163W | **2.3** | Protein of unknown function. |  | |
| *HIR1* | YBL008W | **2.3** | Non-essential transcriptional corepressor . |  | |
| *INP51* | YIL002C | **2.3** | Phosphatidylinositol 4,5-bisphosphate 5-phosphatase. |  | |
| *LDB7* | YBL006C | **2.3** | Protein of unknown function. |  | |
| *MRPL49* | YJL096W | **2.3** | Mitochondrial ribosomal protein of the large subunit. |  | |
| *NNF1* | YJR112W | **2.3** | Essential component of the MIND kinetochore complex. |  | |
| *PRI1* | YIR008C | **2.3** | Subunit of DNA primase. |  |  |
| *QDR3* | YBR043C | **2.3** | Multidrug transporter. |  |  |
| *RCR1* | YBR005W | **2.3** | Protein of the endoplasmic reticulum membrane. |  | |
| *ROT2* | YBR229C | **2.3** | Glucosidase II catalytic subunit. |  | |
| *SEC18* | YBR080C | **2.3** | ATPase. |  | |
| *SIF2* | YBR103W | **2.3** | WD40 repeat-containing subunit of the Set3C histone deacetylase complex. |  | |
| *SMC3* | YJL074C | **2.3** | Subunit of the multiprotein cohesin complex. |  | |
| *TAO3* | YIL129C | **2.3** | Protein involved in cell morphogenesis and proliferation. |  | |
| *TDP1* | YBR223C | **2.3** | Tyrosyl-DNA Phosphodiesterase I. |  | |
| *TIM44* | YIL022W | **2.3** | Peripheral mitochondrial membrane protein . |  | |
| *TRS20* | YBR254C | **2.3** | Subunit of the transport protein particle (TRAPP) complex. |  | |
| *UBC8* | YEL012W | **2.3** | Ubiquitin-conjugating enzyme. |  | |
| *URA8* | YJR103W | **2.3** | Minor CTP synthase isozyme (see also URA7). |  | |
| *VPS35* | YJL154C | **2.3** | Subunit of the membrane-associated retromer complex. |  | |
| *YBR219C* | YBR219C | **2.3** | Hypothetical protein. |  |  |
| *YJL118W* | YJL118W | **2.3** | Hypothetical protein. |  |  |
| *YJR111C* | YJR111C | **2.3** | Hypothetical protein. |  |  |
| *YOL007C* | YOL007C | **2.3** | Structural component of the chitin synthase 3 complex |  | |
| *COQ1* | YBR003W | **2.26** | Hexaprenyl pyrophosphate synthetase. |  | |
| *CYC1* | YJR048W | **2.26** | Cytochrome c, isoform 1. |  |  |
| *MRPL16* | YBL038W | **2.26** | Mitochondrial ribosomal protein of the large subunit |  | |
| *PET112* | YBL080C | **2.26** | Protein required for mitochondrial translation |  | |
| *PTC4* | YBR125C | **2.26** | Cytoplasmic type 2C protein phosphatase. |  | |
| *RTT101* | YJL047C | **2.26** | Cullin subunit of a Roc1p-dependent E3 ubiquitin ligase complex. |  | |
| *STF1* | YDL130W-A | **2.26** | Protein involved in regulation of the mito F1F0-ATP synthase. |  | |
| *STU1* | YBL034C | **2.26** | Component of the mitotic spindle. |  | |
| *YBR014C* | YBR014C | **2.26** | Hypothetical protein. |  |  |
| *YIL015C-A* | YIL015C-A | **2.26** | Hypothetical protein. |  |  |
| *YJL049W* | YJL049W | **2.26** | Hypothetical protein. |  |  |
| *YLR297W* | YLR297W | **2.26** | Hypothetical protein. |  |  |
| *ARG2* | YJL071W | **2.22** | Acetylglutamate synthase (glutamate N-acetyltransferase). |  | |
| *EPS1* | YIL005W | **2.22** | Pdi1p (protein disulfide isomerase)-related protein. |  | |
| *FLR1* | YBR008C | **2.22** | Plasma membrane multidrug transporter. |  | |
| *IML1* | YJR138W | **2.22** | Protein of unknown function. |  | |
| *IML2* | YJL082W | **2.22** | Protein of unknown function. |  | |
| *IMP2'* | YMR035W | **2.22** | Transcriptional activator . |  | |
| *KGD1* | YIL125W | **2.22** | Component of the mito alpha-ketoglutarate dehydrogenase complex. |  | |
| *KTR3* | YBR205W | **2.22** | Putative alpha-1,2-mannosyltransferase. |  | |
| *MAD3* | YJL013C | **2.22** | Component of the spindle-assembly checkpoint complex. |  | |
| *MBA1* | YBR185C | **2.22** | Protein involved in assembly of mitochondrial respiratory complexes. |  | |
| *MGA2* | YIR033W | **2.22** | ER membrane protein involved. |  | |
| *MSC1* | YML128C | **2.22** | Protein of unknown function. |  | |
| *PDX3* | YBR035C | **2.22** | Pyridoxine (pyridoxamine) phosphate oxidase. |  | |
| *RIB5* | YBR256C | **2.22** | Riboflavin synthase. |  |  |
| *RPN4* | YDL020C | **2.22** | Transcription factor; stimulates expression of proteasome genes. |  | |
| *SLT2* | YHR030C | **2.22** | Serine/threonine MAP kinase . |  | |
| *SRO77* | YBL106C | **2.22** | Protein with roles in exocytosis and cation homeostasis. |  | |
| *STH1* | YIL126W | **2.22** | ATPase component of the ATP-dependent RSC chromatin remodeling complex. |  | |
| *SUR1* | YPL057C | **2.22** | Probable catalytic subunit of a mannosylinositol phosphorylceramide synthase. |  | |
| *TBS1* | YBR150C | **2.22** | Probable Zn-finger protein. |  |  |
| *YIL067C* | YIL067C | **2.22** | Hypothetical protein. |  |  |
| *YPL221W* | YPL221W | **2.22** | Protein of unknown function. |  | |
| *ASF1* | YJL115W | **2.18** | Nucleosome assembly factor. |  | |
| *ATG19* | YOL082W | **2.18** | Protein involved in the cytoplasm-to-vacuole targeting pathway. |  | |
| *ATG27* | YJL178C | **2.18** | Type II membrane protein, binds PI 3-phosphate. |  | |
| *BET4* | YJL031C | **2.18** | Alpha subunit of Type II geranylgeranyltransferase. |  | |
| *CIT2* | YCR005C | **2.18** | Citrate synthase. |  |  |
| *CLN2* | YPL256C | **2.18** | G1 cyclin involved in regulation of the cell cycle. |  | |
| *CSG2* | YBR036C | **2.18** | Endoplasmic reticulum membrane protein. |  | |
| *EAF6* | YJR082C | **2.18** | Esa1p-associated factor. |  |  |
| *ECM15* | YBL001C | **2.18** | Protein of unknown function. |  | |
| *GEA1* | YJR031C | **2.18** | Guanine nucleotide exchange factor for ADP ribosylation factors. |  | |
| *GLG2* | YJL137C | **2.18** | Self-glucosylating initiator of glycogen synthesis. |  | |
| *ISY1* | YJR050W | **2.18** | Component of the spliceosome complex . |  | |
| *OPY1* | YBR129C | **2.18** | Protein of unknown function. |  | |
| *PPS1* | YBR276C | **2.18** | Protein phosphatase. |  | |
| *RAD16* | YBR114W | **2.18** | Protein that recognizes and binds damaged DNA in an ATP-dependent manner. |  | |
| *RCY1* | YJL204C | **2.18** | F-box protein . |  | |
| *RPS14B* | YJL191W | **2.18** | Ribosomal protein 59 of the small subunit. |  | |
| *SCS22* | YBL091C-A | **2.18** | Protein involved in regulation of phospholipid metabolism. |  | |
| *SCT1* | YBL011W | **2.18** | Glycerol 3-phosphate/dihydroxyacetone phosphate dual acyltransferase. |  | |
| *SLX1* | YBR228W | **2.18** | Subunit of a complex that hydrolyzes 5' branches from dsDNA. |  | |
| *SWD1* | YAR003W | **2.18** | Subunit of the COMPASS (Set1C) complex. |  | |
| *TFC1* | YBR123C | **2.18** | ssubunit of the RNA pol III transcription initiation factor complex . |  | |
| *YBR096W* | YBR096W | **2.18** | Hypothetical protein. |  |  |
| *YBR255W* | YBR255W | **2.18** | Protein of unknown function. |  | |
| *YDL025C* | YDL025C | **2.18** | Protein of unknown function. |  | |
| *YIL077C* | YIL077C | **2.18** | Hypothetical protein. |  |  |
| *YIR036C* | YIR036C | **2.18** | Hypothetical protein. |  |  |
| *YJL181W* | YJL181W | **2.18** | Hypothetical protein. |  |  |
| *YJR085C* | YJR085C | **2.18** | Hypothetical protein. |  |  |
| *ARP3* | YJR065C | **2.14** | Essential component of the Arp2/3 complex. |  | |
| *ATG12* | YBR217W | **2.14** | Protein that becomes conjugated to Atg5p. |  | |
| *ATH1* | YPR026W | **2.14** | Vacuolar acid trehalase. |  |  |
| *BSD2* | YBR290W | **2.14** | Heavy metal ion homeostasis protein. |  | |
| *CCT7* | YJL111W | **2.14** | Subunit of the cytosolic chaperonin Cct ring complex. |  | |
| *CSH1* | YBR161W | **2.14** | Probable catalytic subunit of a mannosylinositol phosphorylceramide (MIPC) synthase. |  | |
| *GSH1* | YJL101C | **2.14** | Gamma glutamylcysteine synthetase. |  | |
| *GTT2* | YLL060C | **2.14** | Glutathione S-transferase capable of homodimerization. |  | |
| *HAM1* | YJR069C | **2.14** | Protein of unknown function that is involved in DNA repair. |  | |
| *HSM3* | YBR272C | **2.14** | Protein of unknown function. |  | |
| *IDH2* | YOR136W | **2.14** | Subunit of mitochondrial NAD(+)-dependent isocitrate dehydrogenase. |  | |
| *ILM1* | YJR118C | **2.14** | Protein of unknown function. |  | |
| *KNS1* | YLL019C | **2.14** | Protein kinase of unknown function. |  | |
| *ORC2* | YBR060C | **2.14** | Subunit of the origin recognition complex. |  | |
| *POA1* | YBR022W | **2.14** | Phosphatase that is highly specific for ADP-ribose 1''-phosphate. |  | |
| *PRY1* | YJL079C | **2.14** | Protein of unknown function. |  | |
| *PTH2* | YBL057C | **2.14** | One of two mitochondrially-localized peptidyl-tRNA hydrolases. |  | |
| *RIB1* | YBL033C | **2.14** | GTP cyclohydrolase II. |  |  |
| *RSM7* | YJR113C | **2.14** | Mitochondrial ribosomal protein of the small subunit. |  | |
| *SNC1* | YAL030W | **2.14** | Vesicle membrane receptor protein (v-SNARE) . |  | |
| *SSC1* | YJR045C | **2.14** | Mitochondrial matrix ATPase. |  | |
| *YAP5* | YIR018W | **2.14** | Basic leucine zipper (bZIP) transcription factor. |  | |
| *YIA6* | YIL006W | **2.14** | Pvruvate transporter of the mitochondrial inner membrane. |  | |
| *YIR003W* | YIR003W | **2.14** | Hypothetical protein. |  |  |
| *YJL171C* | YJL171C | **2.14** | Hypothetical protein. |  |  |
| *YJL199C* | YJL199C | **2.14** | hypothetical protein. |  |  |
| *YNL158W* | YNL158W | **2.14** | Essential protein required for maturation of Gas1p and Pho8p. |  | |
| *ACH1* | YBL015W | **2.11** | Acetyl-coA hydrolase. |  |  |
| *ARA1* | YBR149W | **2.11** | Large subunit of NADP+ dependent arabinose dehydrogenase. |  | |
| *BCY1* | YIL033C | **2.11** | Regulatory subunit of the cyclic AMP-dependent protein kinase. |  | |
| *CSM2* | YIL132C | **2.11** | Protein required for accurate chromosome segregation in meiosis |  | |
| *ERV15* | YBR210W | **2.11** | Protein of unknown function. |  | |
| *HAL5* | YJL165C | **2.11** | Putative protein kinase. |  |  |
| *HIS5* | YIL116W | **2.11** | Histidinol-phosphate aminotransferase. |  | |
| *MPH1* | YIR002C | **2.11** | Member of the DEAH family of helicases. |  | |
| *MUM2* | YBR057C | **2.11** | Cytoplasmic protein essential for meiotic DNA replication and sporulation. |  | |
| *NTC20* | YBR188C | **2.11** | Member of a complex that binds to the spliceosome. |  | |
| *SEC17* | YBL050W | **2.11** | Peripheral membrane protein required for vesicular transport. |  | |
| *TPS1* | YBR126C | **2.11** | Synthase subunit of trehalose-6-phosphate synthase/ phosphatase complex. |  | |
| *VPS15* | YBR097W | **2.11** | Myristoylated serine/threonine protein kinase. |  | |
| *YBR220C* | YBR220C | **2.11** | Hypothetical protein. |  |  |
| *YBR284W* | YBR284W | **2.11** | Hypothetical protein. |  |  |
| *YIL023C* | YIL023C | **2.11** | Hypothetical protein. |  |  |
| *YIL055C* | YIL055C | **2.11** | Hypothetical protein. |  |  |
| *YJL123C* | YJL123C | **2.11** | Hypothetical protein. |  |  |
| *YPT10* | YBR264C | **2.11** | GTP binding protein that contains the PEST signal sequence. |  | |
| *ABF1* | YKL112W | **2.07** | DNA binding protein with possible chromatin-reorganizing activity. |  | |
| *ARL1* | YBR164C | **2.07** | Soluble GTPase with a role in regulation of membrane traffic. |  | |
| *ATP3* | YBR039W | **2.07** | Gamma subunit of the F1 sector of mito F1F0 ATP synthase. |  | |
| *CAF17* | YJR122W | **2.07** | Mitochondrial protein; interacts with Ccr4p in the 2-hybrid system. |  | |
| *CPA2* | YJR109C | **2.07** | Large subunit of carbamoyl phosphate synthetase. |  | |
| *DJP1* | YIR004W | **2.07** | Cytosolic J-domain-containing protein. |  | |
| *DUR1,2* | YBR208C | **2.07** | Urea amidolyase. |  |  |
| *GPG1* | YGL121C | **2.07** | Proposed gamma subunit of the heterotrimeric G protein. |  | |
| *MNS1* | YJR131W | **2.07** | Alpha-1,2-mannosidase involved in ER quality control. |  | |
| *PET191* | YJR034W | **2.07** | Protein required for assembly of cytochrome c oxidase. |  | |
| *PRP21* | YJL203W | **2.07** | Subunit of the SF3a splicing factor complex. |  | |
| *RRN6* | YBL014C | **2.07** | involved in the transcription of 35S rRNA genes. |  | |
| *RRN7* | YJL025W | **2.07** | involved in the transcription of 35S rRNA genes. |  | |
| *SEF1* | YBL066C | **2.07** | Putative transcription factor. |  | |
| *SNF5* | YBR289W | **2.07** | One subunit of the SWI/SNF chromatin remodeling complex . |  | |
| *STR2* | YJR130C | **2.07** | Cystathionine gamma-synthase. |  | |
| *SYS1* | YJL004C | **2.07** | Integral membrane protein of the Golgi. |  | |
| *TAD2* | YJL035C | **2.07** | Subunit of tRNA-specific adenosine-34 deaminase. |  | |
| *ULP2* | YIL031W | **2.07** | Peptidase that deconjugates Smt3/SUMO-1 peptides. |  | |
| *VTC4* | YJL012C | **2.07** | Vacuolar membrane protein. |  | |
| *YBR075W* | YBR075W | **2.07** | Putative metalloprotease. |  |  |
| *YBR235W* | YBR235W | **2.07** | Hypothetical protein. |  |  |
| *YIL151C* | YIL151C | **2.07** | Hypothetical protein. |  |  |
| *YPL088W* | YPL088W | **2.07** | Putative aryl alcohol dehydrogenase. |  | |
| *ARN2* | YHL047C | **2.03** | Transporter. |  |  |
| *GWT1* | YJL091C | **2.03** | Protein involved in the inositol acylation. |  | |
| *IRA1* | YBR140C | **2.03** | GTPase-activating protein. |  | |
| *LYS12* | YIL094C | **2.03** | Homo-isocitrate dehydrogenase. |  | |
| *MRPL37* | YBR268W | **2.03** | Mitochondrial ribosomal protein of the large subunit. |  | |
| *MRS3* | YJL133W | **2.03** | Mitochondrial iron transporter of the mitochondrial carrier family. |  | |
| *OPT1* | YJL212C | **2.03** | Plasma membrane transporter. |  | |
| *POP7* | YBR167C | **2.03** | Subunit of both RNase MRP. |  | |
| *TAF5* | YBR198C | **2.03** | Subunit (90 kDa) of TFIID and SAGA complexes. |  | |
| *TAT1* | YBR069C | **2.03** | Amino acid transport protein for several aminoacids. |  | |
| *URB2* | YJR041C | **2.03** | Nucleolar protein required for metabolism of the rRNA primary transcript. |  | |
| *YIL137C* | YIL137C | **2.03** | Putative metalloprotease. |  |  |
| *ATP14* | YLR295C | **2.00** | Subunit h of the F0 sector of mitochondrial F1F0 ATP synthase. |  | |
| *CYC8* | YBR112C | **2.00** | General transcriptional co-repressor. |  | |
| *ERV2* | YPR037C | **2.00** | Flavin-linked sulfhydryl oxidase. |  | |
| *HYR1* | YIR037W | **2.00** | Thiol peroxidase. |  | |
| *PDR3* | YBL005W | **2.00** | Transcriptional activator ofthe pleiotropic drug resistance network. |  | |
| *PYC1* | YGL062W | **2.00** | Pyruvate carboxylase isoform. |  | |
| *RFA3* | YJL173C | **2.00** | Subunit of heterotrimeric Replication Factor A (RF-A). |  | |
| *SCP160* | YJL080C | **2.00** | Essential RNA-binding G protein effector of mating response. |  | |
| *SEC66* | YBR171W | **2.00** | Non-essential subunit of Sec63 complex. |  | |
| *SLM1* | YIL105C | **2.00** | Phosphoinositide PI4,5P(2) binding protein. |  | |
| *TIM17* | YJL143W | **2.00** | Essential constituent of the mitochondrial inner membrane presequence translocase. |  | |
| *YJR030C* | YJR030C | **2.00** | Hypothetical protein. |  |  |
| *YJR098C* | YJR098C | **2.00** | Hypothetical protein. |  |  |
| *YJR119C* | YJR119C | **2.00** | Hypothetical protein. |  |  |
| *YOX1* | YML027W | **2.00** | Homeodomain-containing transcriptional repressor. |  | |
| *ARE2* | YNR019W | **0.50** | Acyl-CoA:sterol acyltransferase, isozyme of Are1p. |  | |
| *FUS1* | YCL027W | **0.50** | Membrane protein localized to the shmoo tip. |  | |
| *GSY1* | YFR015C | **0.50** | Glycogen synthase with similarity to Gsy2p. |  | |
| *LOC1* | YFR001W | **0.50** | Nuclear protein involved in asymmetric localization of ASH1 mRNA. |  | |
| *SVL3* | YPL032C | **0.50** | Protein of unknown function |  | |
| *GCV1* | YDR019C | **0.49** | T subunit of the mitochondrial glycine decarboxylase complex. |  | |
| *MET6* | YER091C | **0.49** | Cobalamin-independent methionine synthase. |  | |
| *DIP5* | YPL265W | **0.47** | Dicarboxylic amino acid permease. |  | |
| *PRM6* | YML047C | **0.47** | Pheromone-regulated protein. |  | |
| *QDR2* | YIL121W | **0.47** | Multidrug transporter. |  | |
| *HXK1* | YFR053C | **0.46** | Hexokinase isoenzyme 1. |  |  |
| *HPT1* | YDR399W | **0.45** | Dimeric hypoxanthine-guanine phosphoribosyltransferase. |  | |
| *AGA1* | YNR044W | **0.44** | Anchorage subunit of a-agglutinin of a-cells. |  | |
| *MUC1* | YIR019C | **0.44** | GPI-anchored cell surface glycoprotein. |  | |
| *PNS1* | YOR161C | **0.44** | Protein of unknown function. |  | |
| *PRM4* | YPL156C | **0.44** | Pheromone-regulated protein. |  | |
| *FET4* | YMR319C | **0.42** | Low-affinity Fe(II) transporter of the plasma membrane |  | |
| *YCL065W* | YCL065W | **0.41** | Dubious open reading frame. |  | |
| *YML018C* | YML018C | **0.41** | Hypothetical protein |  |  |
| *PRM7* | YDL039C | **0.41** | Pheromone-regulated protein. |  | |
| *FIG2* | YCR089W | **0.4** | Cell wall adhesin. |  |  |
| *PRM1* | YNL279W | **0.4** | Pheromone-regulated multispanning membrane protein . |  | |
| *TIR1* | YER011W | **0.4** | Cell wall mannoprotein. |  | |
| *FIG1* | YBR040W | **0.39** | Integral membrane protein required for efficient mating. |  | |
| *LAC1* | YKL008C | **0.39** | Ceramide synthase component. |  | |
| *TDH1* | YJL052W | **0.38** | Glyceraldehyde-3-phosphate dehydrogenase. |  | |
| *MF(ALPHA)2* | YGL089C | **0.35** | Mating pheromone alpha-factor. |  | |
| *PRM3* | YPL192C | **0.34** | Pheromone-regulated protein required for karyogamy. |  | |
| *YDL187C* | YDL187C | **0.34** | Questionable ORF. |  |  |
| *RPS22B* | YLR367W | **0.32** | Protein component of the small (40S) ribosomal subunit. |  | |
| *ISF1* | YMR081C | **0.31** | Serine-rich, hydrophilic protein. |  | |
| *FRE4* | YNR060W | **0.29** | Ferric reductase. |  |  |
| *PLB2* | YMR006C | **0.29** | Phospholipase B (lysophospholipase) . |  | |
| *MMP1* | YLL061W | **0.28** | High-affinity S-methylmethionine permease. |  | |
| *GPH1* | YPR160W | **0.25** | Non-essential glycogen phosphorylase. |  | |
| *YOR343C* | YOR343C | **0.23** | Hypothetical protein. |  |  |
| *ZRT1* | YGL255W | **0.20** | High-affinity zinc transporter of the plasma membrane. |  | |
| *RPS9A* | YPL081W | **0.19** | Protein component of the small (40S) ribosomal subunit. |  | |
| *CCW12* | YLR110C | **0.17** | Cell wall protein. |  |  |
| *YAR068W* | YAR068W | **0.06** | Putative membrane protein. |  |  |
| *HXT4* | YHR092C | **0.05** | High-affinity glucose transporter. |  | |
|  |  |  |  |  |  |

**Supplementary Table 1**

ORFs differentially expressed in mutant *ccw12*Δ.
