# Supplementary material for: The genetic interaction network of CCW12, a Saccharomyces cerevisiae gene required for cell wall integrity during budding and formation of mating projections
Source: BMC Genomics. 2011 Feb 14;12:107. doi: 10.1186/1471-2164-12-107 (PMC3049148; doi:10.1186/1471-2164-12-107)
Supplement: Additional file 3 — Northern blot analysis of ECM13 and PIR3 expression in ccw12Δ cells [file 1471-2164-12-107-S3.PDF]

Supplementary Figure 1

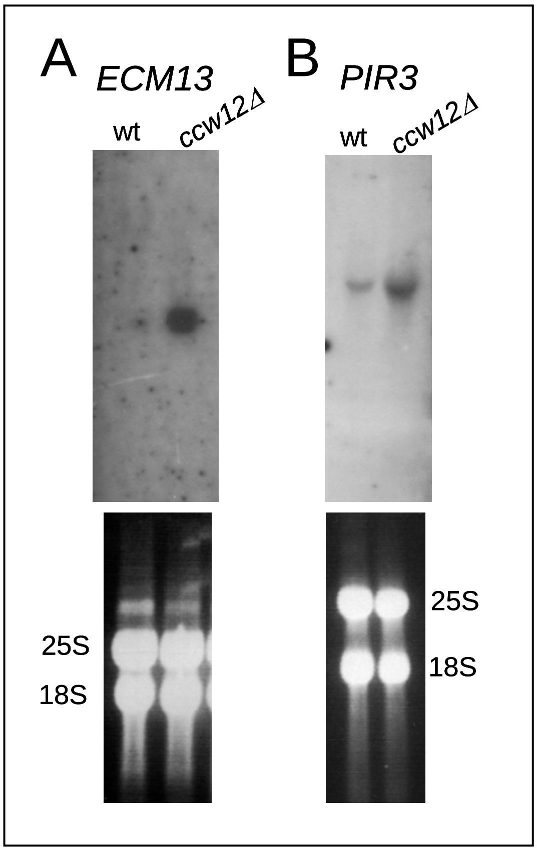

Supplementary Figure 1 – Northern blot analysis

Yeast strains SEY6210 (wt) and MEY12A (*ccw12*D) were grown in liquid YPD medium to early log phase. Total RNA was isolated and probed with an *ECM13*-specific **(A)** and a *PIR3*-specific **(B)** DNA probes, respectively, using standard procedures.

The 679 bp *ECM13*-specific DNA probe was prepared by PCR using oligonucleotides 539 (caaagtacgctccaagctg) and 540 (atcgtcgacatcgctactag). The 628 bp *PIR3*-specific DNA probe was prepared by PCR using oligonucleotides 545 (tgatggtcaagtacaggctg) and 546 (gagctaagttaccttctggg). **(A, B)** The *upper panel* shows a Northern blot, using the 32P-labeled gene-specific DNA probe. Each lane was loaded with 25 µg of total RNA. The *lower panel* shows the identical gel after electrophoresis (without blotting), and the gel was stained with acridine orange. This panel reveals that roughly equal amounts of intact 25 S and 16 S ribosomal RNA were present in each sample.
